# A review and synthesis of the first 20 years of PET and fMRI studies of heard speech, spoken language and reading

**DOI:** 10.1016/j.neuroimage.2012.04.062

**Published:** 2012-08-15

**Authors:** Cathy J. Price

**Affiliations:** Wellcome Trust Centre for Neuroimaging, UCL, London, WC1N 3BG, UK

**Keywords:** PET, fMRI, Language, Auditory speech, Reading, Comprehension, Speech production

## Abstract

The anatomy of language has been investigated with PET or fMRI for more than 20 years. Here I attempt to provide an overview of the brain areas associated with heard speech, speech production and reading. The conclusions of many hundreds of studies were considered, grouped according to the type of processing, and reported in the order that they were published. Many findings have been replicated time and time again leading to some consistent and undisputable conclusions. These are summarised in an anatomical model that indicates the location of the language areas and the most consistent functions that have been assigned to them. The implications for cognitive models of language processing are also considered. In particular, a distinction can be made between processes that are localized to specific structures (e.g. sensory and motor processing) and processes where specialisation arises in the distributed pattern of activation over many different areas that each participate in multiple functions. For example, phonological processing of heard speech is supported by the functional integration of auditory processing and articulation; and orthographic processing is supported by the functional integration of visual processing, articulation and semantics. Future studies will undoubtedly be able to improve the spatial precision with which functional regions can be dissociated but the greatest challenge will be to understand how different brain regions interact with one another in their attempts to comprehend and produce language.

## Introduction

In the last 20 years, there has been an explosion of research into the neural basis of language processing. This has clearly established that spoken and written language relies on concurrent activation in multiple brain areas. The location of these regions has been identified with functional neuroimaging techniques such as Positron Emission Tomography (PET) and functional Magnetic Resonance Imaging (fMRI) that measure hemodynamic changes, while the timing of brain activity during language processing has been identified using electromagnetic techniques such as magnetoencephalography (MEG) and electroencephalography (EEG). Although it is the combination of these spatial and temporal measurements that is needed to provide a mechanistic account of language (Demonet and Thierry, 2001), the current review focuses only on the localisation of language and reading areas with PET and fMRI.

Prior to the availability of functional imaging techniques, our understanding of language in the brain rested on studies of impairments in patients with brain damage or patients undergoing electrical stimulation during neurosurgery. The most popular neural model of language was based on the writings of Broca, Wernicke and Lichtheim at the end of the 19th Century and Geschwind in the mid 20th Century (Broca, 1861; Wernicke, 1874; Lichtheim, 1885; Geschwind, 1965). Auditory speech recognition was localized to the left posterior temporal cortex (Wernicke's area); speech production (motor word representations) was localized to the left posterior inferior frontal cortex (Broca's area); and visual word recognition was localized to the left angular gyrus (Dejerine, 1891). One of the major limitations with this classical neurological model is that it does not indicate how single words are combined into meaningful sentences. This was highlighted in the latter half of the 20th Century, when it was noted that although Broca's aphasics could comprehend heard speech based on semantic content, they had difficulty comprehending sentences that depended on complex syntactic analysis (Caramazza and Zurif, 1976). It was also noted that circumscribed damage to Broca's area only resulted in temporary speech production difficulties and that permanent speech production difficulties were observed when frontal lobe damage extended into the insula and parietal regions in patients with left middle cerebral artery infarcts (Mohr et al., 1978).

The neurological model of language also failed to explain why those with reading difficulties might have a selective impairment of the ability to read whole words with atypical spellings such as “yacht” or, conversely, a selective impairment of the ability to read nonwords with no meaning such as “yatched” (Marshall and Newcombe, 1973). Such observations suggested that there are two or more different pathways to speech output from text. The complexity of language processing and the importance of many regions outside Broca's and Wernicke's territories were therefore well appreciated prior to the availability of functional imaging techniques such as PET and fMRI.

### Early PET studies

**Fig. 1:**
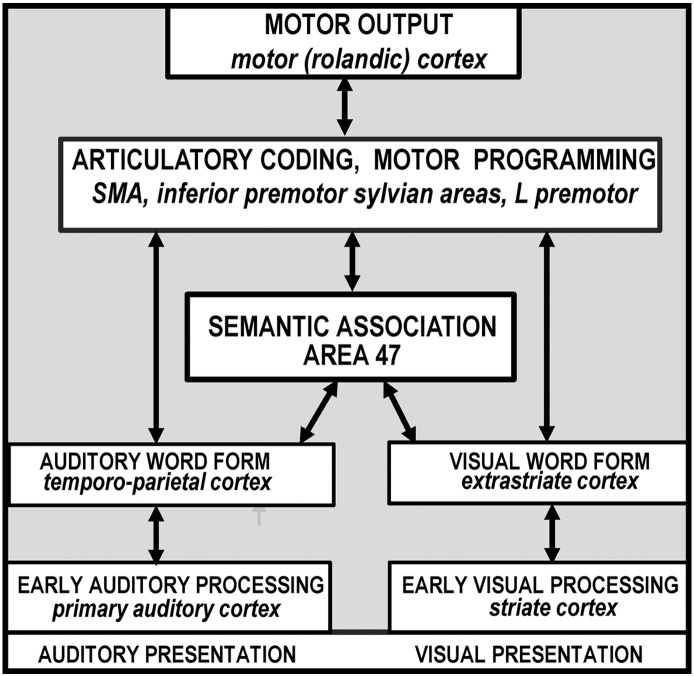
Functional–anatomical model proposed by Petersen et al. (1988, 1989).

The landmark of functional imaging study of auditory and visual word processing was published in 1988 by Petersen and colleagues who used PET to identify the brain areas activated when healthy participants were presented with auditory or visual single words and were instructed either to view them passively, repeat them or generate a verb that was related to the heard or seen noun (e.g. “eat” in response to “cake”). On the basis of the results and other deductions, the authors concluded that (i) auditory word forms were processed in the left temporoparietal cortex, (ii) visual word forms were processed in the left extrastriate cortex, (iii) semantic associations involved the left ventral prefrontal cortex, (iv) word generation involved the dorsolateral prefrontal cortex; (v) general response selection involved the anterior cingulate; (vi) articulatory coding and motor programming involved the left ventral premotor cortex, left anterior insula (referred to as the buried sylvian cortex) and supplementary motor cortex (SMA) and (vii) motor execution involved the rolandic cortex (the posterior part of the precentral gyrus bordering the central sulcus). Together the results provided a new anatomical model of lexical processing (Petersen et al., 1988; Petersen et al., 1989) that is illustrated in Fig. 1. The key features of this model were the inclusion of a small number of discrete areas with multiple parallel routes between localized sensory-specific, phonological, articulatory and semantic-coding areas.

Shortly after this study, the same authors published a PET study of reading that compared regional activation in response to visual words and word-like stimuli (Petersen et al., 1990). The association of the left medial extrastriate cortex with visual word form processing was confirmed because this area was activated by visually presented words and pseudowords that obey English spelling rules but was not activated by unfamiliar strings of letters or letter-like forms. In addition, activation in the left frontal cortex which had been associated with semantic processing during word generation (Petersen et al., 1988; Petersen et al., 1989) was found to be more activated by passive presentation of words than pseudowords. Thus Petersen et al. (1990) were able to distinguish high-level visual and semantic computations on single words and describe the underlying anatomy.

In 1991, Petersen and colleagues' early findings were replicated and extended. Wise et al. (1991a) found that the response in the left posterior superior temporal cortex (Wernicke's area) dissociated from that in other left and right superior temporal regions because only activation in the left posterior temporal area was independent of the rate of presentation of the auditory input. The importance of considering how frontal and temporal lobes interacted was highlighted by Frith et al. (1991) who demonstrated that when words were produced without stimuli (i.e. verbal fluency) activation increased in the left dorsolateral prefrontal cortex but decreased in superior temporal cortices, whereas, during lexical decisions on heard words, activation increased in the superior temporal cortices with no change in prefrontal activation. In a follow up paper (Friston et al., 1991), the same authors pursued the emphasis on regional interactions by correlating activation in the superior temporal gyrus and prefrontal cortex and proposing that word representations were distributed in the left superior temporal cortex and word processing in the temporal lobe was modulated by the left dorsolateral prefrontal cortex.

The importance of these early PET studies was far reaching. They illustrated that functional imaging could provide anatomical localization with a precision that far exceeds that attainable with human brain lesion studies. Moreover, the study of healthy subjects avoids possible confounding effects of brain lesions, such as compensatory reorganization of brain function (Haxby et al., 1991; Raichle, 1991; Wise et al., 1991b). Methodological challenges were also well appreciated particularly when the results appeared to contradict classic axioms of language organization. For example, Steinmetz and Seitz (1991) argued that data should not be averaged over subjects because intraoperative stimulation showed diversity in location of language functions and morphometrical imaging studies showed diversity of brain shape and gyral patterns that would be difficult to correct with anatomical normalisation techniques. Many other concerns were succinctly addressed in a review by Petersen and Fiez (1993) who pointed out that functional neuroimaging results should be viewed as evolutionary, rather than revolutionary and that they were most interpretable when they were backed up by supporting data from other studies. For example, after the Petersen et al. (1988), Petersen et al. (1989) observation that the right lateral inferior cerebellum was activated by cognitive rather than sensory motor computations, they demonstrated that damage to the right cerebellum impairs practice related learning and detection of errors (Fiez et al., 1992). Petersen and Fiez (1993) also emphasized that complex language functions were not localized in specific brain regions; they were distributed across networks of regions with each area making a specific contribution to the performance of the task which depends on its connections to other areas in a parallel distributed hierarchy. In this context, understanding the functional anatomy of language cannot be deduced from a single experiment; rather, it requires the integration of results from multiple experiments using multiple techniques.

### Early fMRI studies

The first fMRI studies of language processing were reported by McCarthy et al. (1993), Hinke et al. (1993), Binder et al. (1994a, 1994b, 1995, 1996a, 1996b), Pugh et al. (1996) and Small et al. (1996). The results provided a reassuring demonstration that fMRI could replicate the findings of PET. For example, McCarthy et al. (1993) showed that word generation, relative to repetition, activated the inferior frontal cortices and anterior insula as previously reported using PET (Petersen et al., 1988; Petersen et al., 1989), while Binder et al. (1994b) and Dhankhar et al. (1997) illustrated that increasing the rate of presentation of simple auditory speech stimuli resulted in a monotonically proportional increase in bilateral superior temporal lobe activation, as previously reported using PET (Wise et al., 1991a,1991b; Price et al., 1992). As fMRI became more available, functional imaging studies of language could be conducted on children and females of childbearing age who had previously been excluded because of the risk of the radiation dose involved in PET scanning. As fMRI is a non-invasive procedure, the same subject could be scanned multiple times thereby providing robust data from individual subjects and this opened the way for studies of inter-subject variability (Demb et al., 1995; Huckins et al., 1998; Demonet et al., 2005).

## Methods

### Inclusion criteria for the review

This review focuses on papers, reported between 1992 and 2011, that aimed to identify the brain areas activated when healthy adults were engaged in speech comprehension and production tasks. Papers were selected from PubMed if their titles or abstracts included a specific combination of search words. One search word would be a language process of interest (e.g. semantics, phonology, comprehension, articulation, etc.) while the other search word would be either an imaging term (e.g. fMRI, PET or functional imaging) or a brain region (e.g. SMA, cerebellum). Papers were excluded if: (a) their aim was to infer language processing from brain activation (as opposed to reporting brain activation in response to language processing), (b) their conclusions were about abnormal populations (e.g. in dyslexics, schizophrenics, stroke patients etc.) and (c) they reported activations that varied across normal populations (e.g. due to age, gender, ability etc.). This was not from lack of interest but due to the time frame for writing the review and the overwhelming number of papers (> thousand) that needed to be considered. The papers identified are also influenced by my personal memories of how our knowledge unfolded over the past 20 years, the availability of papers on PubMed, the choice of search terms used by me and the key words used by the authors.

For each paper, I extracted the conclusions stated in the abstract; and made minimal adjustments to the functional and anatomical terms. I only changed terminology that was inconsistent with that used in other studies. For example, the terms Broca's area, BA 44, inferior frontal cortex and pars opercularis have been used interchangeably. I chose pars opercularis to make a clear distinction with other frontal regions, such as the pars orbitalis, pars triangularis and premotor cortex. Contrary to my previous review (Price, 2010) and methodology used in computational meta-analyses (e.g. Turkeltaub et al., 2002; Jobard et al., 2003; Binder et al., 2009; Vigneau et al., 2011), the conclusions are not based on the standard (Talairach or MNI) co-ordinates of peak activations. The disadvantage of not using a co-ordinate based meta-analysis is that the conclusions depend on the subjective interpretation of the authors. However, computational meta-analyses based on reported co-ordinates are also subjective because they depend on the statistical threshold used by the authors, the sensitivity of the paradigm (conditions and number of participants), the precision with which the co-ordinates describe the extent of the activation, and the inclusion criteria of the meta-analysis. The methodology used in the current review has the advantage of being able to report (a) conclusions drawn by authors who controlled the collection of the data; and (b) a historical perspective of how and when the conclusions emerged.

### Organization of the conclusions

The reporting of results is structured around three sections that focus on auditory speech comprehension, speech production and reading. Within the auditory speech section, the subsections focus on auditory responses that do not distinguish speech from nonspeech; auditory responses that are greater for speech than nonspeech; and comprehension of speech at the word and sentence level. Within the speech production section, the subsections focus on word retrieval, covert articulatory planning, overt articulation and post-articulatory sensorimotor feedback. Within the visual word processing section, the subsections focus on responses that distinguish visual words from other visual stimuli and differences between the lexical and sublexical mapping of orthography (letter combinations) to phonology (sounds).

To demonstrate the progressive steps in the evolution of our knowledge, I have further divided each subsection into 4 time era according to the year of publication. These are (i) 1992–1996 when PET was in its heyday and the contribution of fMRI was being validated; (ii) 1997–2001 when fMRI was taking over; (iii) 2002–2006 when there were notable improvements in the spatial definition of language areas; and (iv) 2007–2011 when there were many further refinements as well as a much greater focus on using functional anatomy to test cognitive models of language. Within each of these time era, I have also attempted to dissociate novel conclusions from replicated conclusions.

## Results

**Fig. 2:**
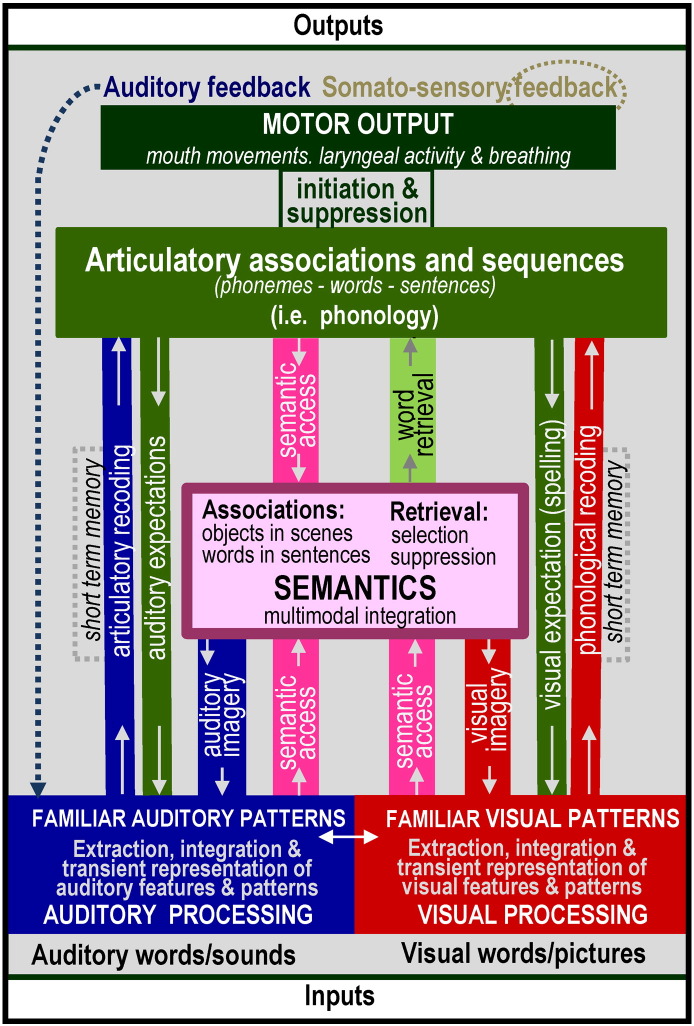
Functional model based on neuroimaging studies of language.

**Fig. 3:**
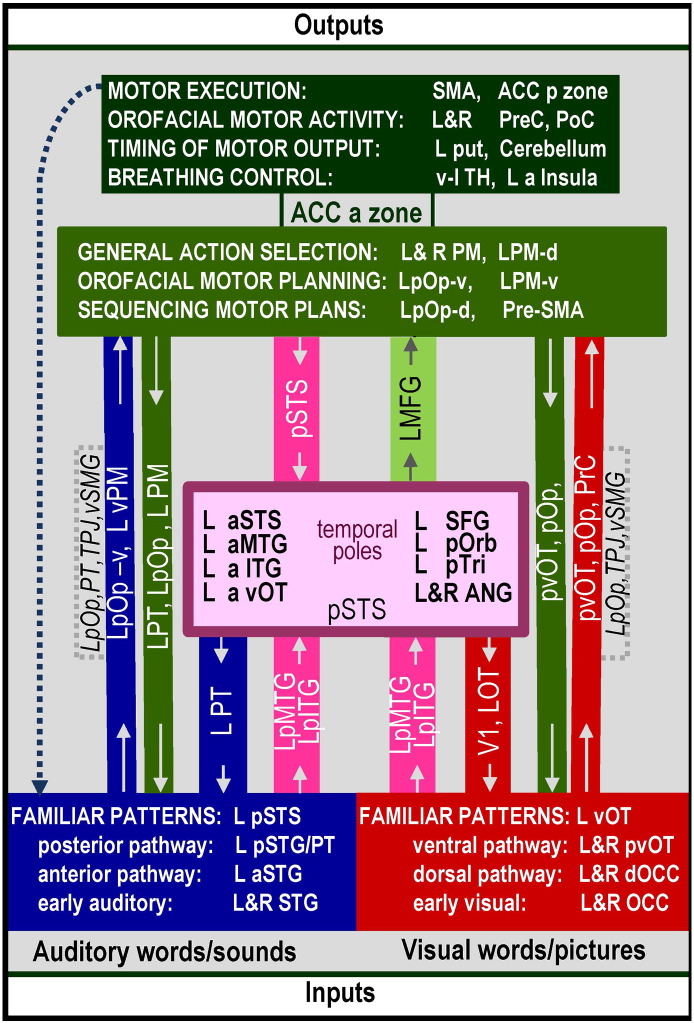
Anatomical model based on neuroimaging studies of language. See Fig. 4 and Table 3 for key to anatomical abbreviations.

**Fig. 4:**
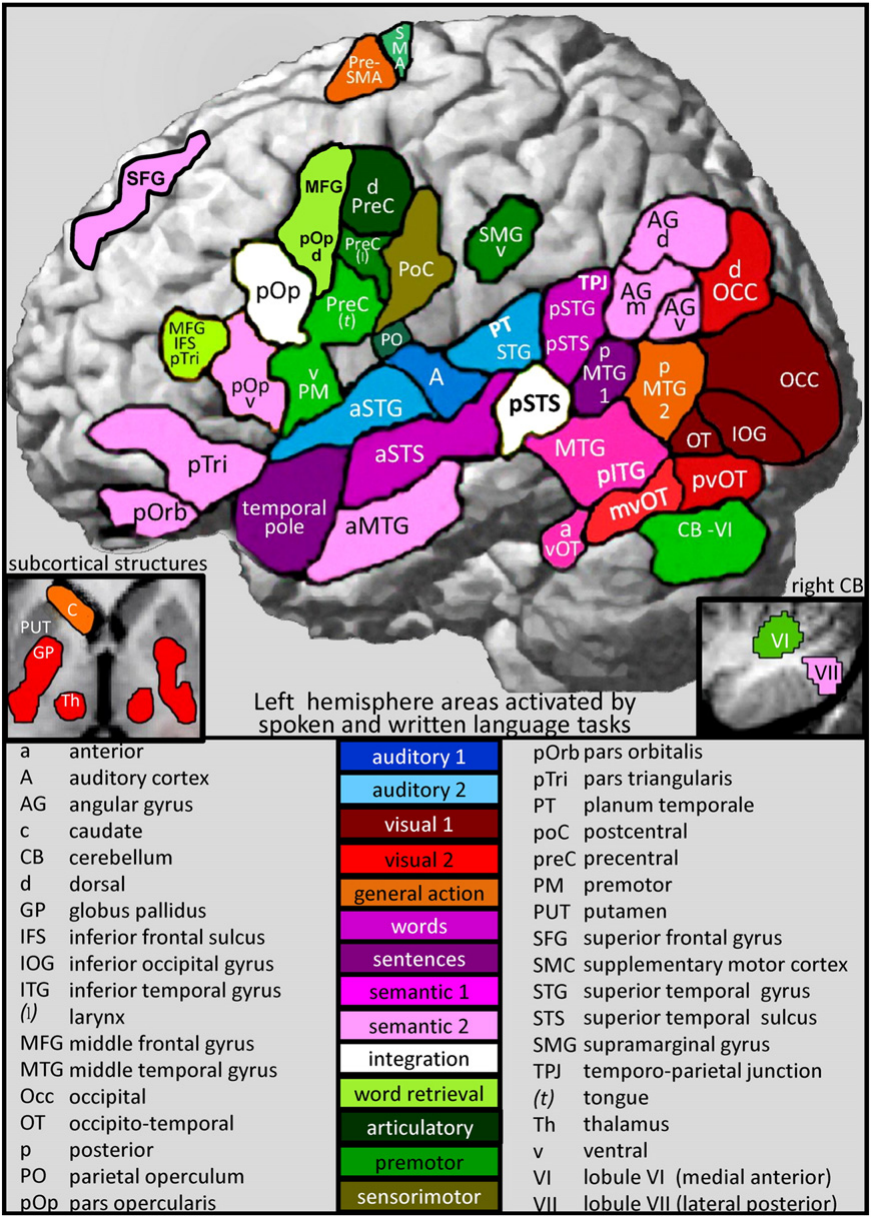
Illustrative sketch of the location of language related activations, based on Price et al. data. This figure was created by overlaying images of activations from many different studies that I have co-authored. Details of the studies can be provided on request to the author. The colours indicate the type of task or processing that caused the activation. The blue areas are activated by auditory stimuli (auditory 1 followed by auditory 2). The red/brown areas are activated by visual stimuli (visual 1 then visual 2). The orange areas are activated by general action selection (hand or speech). The pink and purple areas are involved in different levels of semantic and syntactic processing depending on the task demands. The dark pink areas are sensitive to the semantic content of the stimuli, the light pink areas are those that more activated for semantic than phonological decisions. The light purple areas are activated by spoken and written sentences with the dark purple areas most activated by meaningful and grammatical sentences. The green areas are involved in generating or rehearsing speech. The light green areas are involved in word retrieval, the dark green areas are involved in silent phonological decisions. The khaki green area and PreC/vPM areas are activated by mouth movements during speech. Finally the white areas, corresponding to Broca's area (pOp) and Wernicke's area (pSTS) are involved in both perception and production tasks with familiar stimuli. They may function as convergence zones that receive and send signals to all the other areas involved in perceiving and producing speech. The connections between areas are not shown because we don't yet know enough about how all the areas connect to one another.

The results of the review are reported in relation to the functional–anatomical model from (Petersen et al. 1988; Petersen et al., 1989) which is illustrated in Fig. 1. This was edited to provide more functional terms (Fig. 2) and the anatomical attributions associated with these functions (Fig. 3). Fig. 4 provides a sketch of the left hemisphere anatomical locations of the activations related to different language-related functions, after rendering activations from my own data onto a canonical model of the left hemisphere. Table 1 defines the functional terminology used in Fig. 2. Table 2 provides a historical perspective of the anatomical attributions according to the time era in which they emerged. Table 3 provides the full anatomical names of the abbreviations used in Table 2. Table 4 reverses the inference in Table 2 by listing the anatomical regions in alphabetical order for easy reference to the language functions identified in the review. The studies that contributed to the review and my synthesis of its findings are provided in the Discussion.

## Discussion

### Auditory processing that is common to speech and nonspeech sounds

This section is included because studies of auditory responses that are not specific to speech sounds have provided important clues for understanding how speech processing emerges. For example, “left lateralized” nonspeech processing may be a precursor to left lateralized higher-level language functions.

#### Auditory processing of speech and nonspeech. Time era: 1992–1996

##### Novel findings

One of the most surprising early findings was that the planum temporale (on the dorsal surface of the superior temporal gyri and the ventral surface of the sylvian fissure) showed similar responses to heard speech and tones (Binder et al., 1996a). This finding suggested that both the left and right planum temporale were involved in early auditory processing, which is contrary to the classic view that the left planum temporale was specialised for language (Geschwind and Levitsky, 1968). An equally surprising finding was that the left planum temporale was activated in the absence of any auditory input (McGuire et al., 1996a,b): for example, during silent speech production and when subjects imagined hearing another person's voice (McGuire et al., 1996a). This suggested a special role for the planum temporale in higher-level auditory representations that could be tapped by bottom up processing of auditory inputs or top-down processing of learnt auditory images.

#### Auditory processing of speech and nonspeech. Time era: 1997–2001

##### Extending prior findings

Further evidence was reported that the left planum temporale was involved in silent auditory imagery of speech (Shergill et al., 2001) or when recalling (imagining) the auditory relative to visual associations of a picture of a scene (Wheeler et al., 2000).

##### Novel findings

Functional subdivisions were described within the bilateral auditory cortices (Mirz et al., 1999) with simple auditory stimuli activating the transverse temporal gyri (BA 41), sounds with discontinuous acoustic patterns activating the surrounding superior temporal gyri (BA 42), and sounds with complex spectral intensity, and temporal structures (heard words and music) activating areas that extended into the bilateral superior temporal sulci (BA 21, 22). Zatorre and Belin (2001) also noted that anterior regions in bilateral superior temporal cortex were particularly sensitive to variation in the spectral content of nonverbal stimuli.

Left lateralized responses to nonspeech sounds were identified in the auditory cortex (superior temporal gyri) by Belin et al. (1998) when participants listened to nonverbal sounds containing rapid relative to slow frequency transitions (Belin et al., 1998; Zatorre and Belin, 2001; Giraud et al., 2000). As rapid frequency modulations are a key feature in speech sounds, the observed left lateralization was proposed to contribute to the lateralization of higher-level language functions.

Beyond the temporal lobes, speech and nonspeech sounds activated the left inferior frontal cortex (pars triangularis and pars opercularis) when they needed to be segmented and held in auditory working memory (Burton et al., 2000; Pedersen et al., 2000; Hsieh et al., 2001; Poldrack et al., 2001). This is important for appreciating that left inferior frontal activation during auditory speech processing does not necessarily indicate a speech specific function.

#### Auditory processing of speech and nonspeech. Time era: 2002–2006

##### Extending prior findings

Many studies observed activation in bilateral dorsal superior temporal cortices during early auditory processing of both speech and nonspeech sounds (Booth et al., 2002a, 2002b; Giraud et al., 2004; Hickok et al., 2003; Hickok and Poeppel, 2004; Meyer et al., 2005) with increased activation when acoustic complexity increased (Hwang et al., 2005), the rate of word presentation increased (Noesselt et al., 2003), when the listener had to segregate two simultaneously presented speech sounds (Alain et al., 2005; Scott et al., 2004), for the perception of distorted speech sounds (Davis and Johnsrude, 2003; Meyer et al., 2004), for hearing syllables relative to vowels (Jancke et al., 2002) and for auditory speech in noisy environments (Scott et al., 2004).

Auditory imagery of the sounds was again associated with left lateralised activation in the planum temporale in response to experience with tones (Xu et al., 2006) and visual stimuli (Jancke and Shah, 2004; Pekkola et al., 2006), in the silence that followed familiar music even when there was no instruction to remember the music (Kraemer et al., 2005), when passively viewing finger tapping on the piano following keyboard training (Hasegawa et al., 2004), when producing rhythmic finger sequences that had been learnt with an auditory cue (Bengtsson et al., 2005) and when imagining heard speech, music or environmental sounds in the absence of sound (Aleman et al., 2005; Bunzeck et al., 2005; Zatorre and Halpern, 2005).

The view that left lateralization for heard speech might arise at the level of detecting rapidly changing temporal features (Poeppel, 2003; Hesling et al., 2005) was strengthened by observations that posterior temporal activation was left lateralized for rapid relative to slow frequency transitions (Zaehle et al., 2004; Rimol et al., 2005; Husain et al., 2006) and for broad relative to narrow band speech envelope noises (Giraud et al., 2004; Specht et al., 2005). Alternative hypotheses were that left lateralization in both temporal and inferior frontal areas were related to a top down attentional bias (Davis and Johnsrude, 2003) or auditory-motor processing (Hickok et al., 2003; Okada and Hickok, 2006; Buchsbaum et al., 2005a,b).

Right lateralized auditory responses were observed for environmental sounds (Specht and Reul, 2003; Thierry et al., 2003; Thierry and Price, 2006), non-linguistic vocal sounds like laughter (Belin et al., 2002; Kriegstein and Giraud, 2004; Meyer et al., 2005), and the familiarity of vocalisation (Kriegstein and Giraud, 2004). These right hemisphere responses may help to explain why the perception of prosody in heard speech prosody is associated with the right hemisphere, particularly when the language demands of the task are low (Gandour et al., 2004; Meyer et al., 2004).

#### Auditory processing of speech and nonspeech. Time era: 2007–2011

##### Extending prior findings

Bilateral superior temporal activation was reported for the acoustic analysis of speech and nonspeech sounds (Turkeltaub and Coslett, 2010; Obleser et al., 2007a, 2007b; Dick et al., 2011) and shown to be sensitive to frequency discriminations (Zaehle et al., 2008), familiarity (Raettig and Kotz, 2008; Davis and Gaskell, 2009; Kotz et al., 2010; Vaden et al., 2010), spectral structure and temporal modulation (Britton et al., 2009; Leaver and Rauschecker, 2010).

Left lateralized responses were reported for the discrimination of fast changing verbal and nonverbal sounds in the planum temporale (Elmer et al., 2011a) and for the perceptual interpretation of speech sounds in early auditory areas (Kilian-Hutten et al., 2011). In contrast, right auditory areas were associated with changes of the frequency spectrum (Obleser et al., 2008), categorical perception of familiar musical chords, and the comparison of familiar versus unfamiliar musical sequences (Klein and Zatorre, 2011; Peretz et al., 2009), spectrally rotated speech sounds compared to speech masked by noise (Scott et al., 2009b) and rhythm and intonation in nonspeech (Zhang et al., 2010). This provided further support for the importance of right superior temporal activation in the prosodic processing of speech which relies on detecting variations in the frequency spectrum, rhythm and intonation.

##### Novel perspectives

The association of the planum temporale with auditory imagery during silent production tasks (e.g. silent humming) was replicated (Pa and Hickok, 2008) but complicated by multiple reports that the posterior planum temporale (on the dorsal surface of the posterior superior temporal gyrus) and the surrounding temporoparietal cortex were activated under a wide range of conditions including visual stimuli without auditory stimuli or auditory associations (Meyer et al., 2007). In addition, activation in the planum temporale was reported during phonation and exhalation (Loucks et al., 2007), auditory working memory (Buchsbaum and D'Esposito, 2009; Koelsch et al., 2009; McGettigan et al., 2011) and for syntactic processing of auditory and written sentences (Friederici et al., 2009; Raettig et al., 2010; Richardson et al., 2010).

The multi-functional responses reported in the posterior planum temporale may have resulted from conflating different functional regions (Zheng, 2009; Price, 2010; Isenberg et al., 2011; Tremblay et al., 2011). For example, the practice of functionally defining a region of interest that is commonly activated by auditory inputs and covert articulation (Hickok et al., 2003) might artificially merge distinct auditory and motor regions in the ventral and dorsal surfaces of the sylvian fissure particularly when data are smoothed and averaged over subjects. A second possibility is that, within the same temporoparietal region, there are multiple overlapping functionally distinct cell populations for perception and covert production (Hickok et al., 2009).

A third explanation of common responses to auditory, motor and memory processes in the same anatomical area is that they reflect a common process. For example, activation during auditory working memory, silent motor tasks and sentence processing can all be explained by the demands on auditory imagery. Models of visual processing provide a useful analogy here because it has been shown that early visual cortices (e.g. the calcarine cortex and the lateral occipital cortex) are activated during visual imagery in the absence of visual stimulation (Klein et al., 2000; Lambert et al., 2002; Stokes et al., 2009, 2011) with this early sensory activation sustained by recurrent interactions with higher-level processing areas (Stokes et al., 2009, 2011). Future studies are therefore needed to provide a more precise definition of the functional responses in both the left posterior planum temporale and the left ventral supramarginal gyrus during auditory processing.

#### Auditory processing of speech and nonspeech: 20 year summary

Auditory processing of speech and nonspeech sounds activates the bilateral superior temporal gyri (STG in Table 2 and Fig. 3) that include and surround Heschl's gyri. Three observations relevant to higher-level speech processing have been described. First, left lateralized superior temporal activation during auditory processing has been observed and related to hemispheric differences in the processing of rapidly changing auditory inputs and/or the influence of left lateralized inferior frontal and temporo-parietal activation (LpOp, PT and TPJ in Table 2 and Fig. 3) that is involved when auditory inputs need to be segmented in a meaningful way. Second, the involvement of left inferior frontal and temporo-parietal activation during auditory segmentation tasks might be a consequence of auditory short-term memory, covert articulation or vocalisation mechanisms. Third, the left planum temporale (PT in Table 2 and Fig. 3) on the dorsal surface of the superior temporal gyrus is activated by imagining sounds (in silence) as well as hearing sounds. This suggests that the left planum temporale might be the recipient of top-down processing from higher-order language areas.

### Speech selective auditory responses (= phonological processing)

Speech sound processing refers to the unique dynamic acoustic patterns that can be generated by the human speech production system. Abstract knowledge of familiar speech sounds is referred to as phonological memory and can be accessed from visual stimuli (e.g. text) as well as auditory speech. Although there was an expectation that there would be brain areas that were dedicated to auditory speech processing, none of the areas discussed below turned out to be uniquely activated by speech.

#### Speech selective auditory responses. Time era: 1992–1996

##### Novel findings

Two studies investigated speech processing during decisions on the sound structure of speech relative to nonspeech sounds (e.g. tones) and reported speech-related activation in bilateral secondary auditory cortices and the left posterior inferior frontal cortex (Zatorre et al., 1992; Demonet et al., 1992, 1994a). The interpretation of this speech related activation (Zatorre et al., 1992) was in terms of pre-lexical processing in the bilateral secondary auditory cortices with articulatory recoding (i.e. subvocal articulation of the speech sounds) in the left posterior inferior frontal cortex . However, the comparison of speech and tones in these early studies did not control for auditory complexity or auditory segmentation and, as described in the Auditory processing that is common to speech and nonspeech sounds section above, activation in the bilateral auditory cortices and the left posterior inferior frontal cortex was not specific to speech.

It was also demonstrated that activation for speech depended on task. For example, passive listening tasks were associated with left lateralized activation for words relative to tones in the superior temporal sulcus, middle temporal gyrus and angular gyrus (Binder et al., 1996a), with inferior frontal activation that was more ventral and anterior to that observed during phonetic judgements and auditory repetition (BA 45 vs. BA 44; Price et al., 1996b). The same ventral inferior frontal areas were subsequently associated with semantic analysis (see Speech comprehension section below) which is not controlled during the passive listening tasks.

Consideration was also given to the importance of the ventral part of the left anterior supramarginal gyrus (vSMG) in speech processing. One interpretation was that co-activation in this area and the left posterior inferior frontal cortex was related to articulatory rehearsal during auditory short-term memory tasks (Paulesu et al., 1993) and phonological decisions (Demonet et al., 1994b). The alternative interpretation was that ventral supramarginal gyrus activation was related to stimulus encoding rather than memory mechanisms (Fiez et al., 1996b).

#### Speech selective auditory responses. Time era: 1997–2001

##### Novel findings

The special role of the left posterior temporal lobe in speech processing was re-considered (Celsis et al., 1999; Scott et al., 2000; Wise et al., 2001). Celsis et al. (1999) reported a common response to speech and nonspeech stimuli in the upper surface of the gyrus but Wise et al. (2001) identified a more ventral region in the left lateral posterior superior temporal sulcus (pSTS in Fig. 3) that was activated by both word perception and the retrieval of words from memory (in response to a semantic cue). Wise et al. (2001) therefore proposed that this area is involved in transiently representing the temporally ordered sound structure of both heard words and words retrieved from lexical memory. This function would serve a number of key language tasks such as mimicry, repetition and the long-term acquisition of new words. The key difference between this memory function in pSTS (which is in the sulcus on the lower surface of the superior temporal gyrus) and the posterior planum temporale (which is on the dorsal surface of the superior temporal gyrus) is that pSTS is more involved in speech than nonspeech whereas the planum temporale does not distinguish speech and non-speech (see Auditory processing that is common to speech and nonspeech sounds section).

#### Speech selective auditory responses. Time era: 2002–2006

##### Extending prior findings

The speech selective auditory response in the left posterior superior temporal sulcus was repeatedly demonstrated even when acoustic complexity was controlled (Narain et al., 2003; Giraud et al., 2004; Hugdahl et al., 2003; Benson et al., 2006; Rimol et al., 2006). Further evidence that these speech selective responses relate to familiarity with the patterns of spectral variation (Liebenthal et al., 2003) came from two studies that showed activation in the left posterior temporal gyrus/sulcus increased when sine wave analogues of speech were recognised as speech relative to when the same stimuli were not recognised as speech (Dehaene-Lambertz et al., 2005; Meyer et al., 2005). A contrasting pattern of response was observed in the anterior processing stream where activation was typically observed when acoustic complexity was not controlled (Obleser et al., 2006; Uppenkamp et al., 2006; Binder et al., 2004), even when familiarity and conceptual content were matched by comparing speech to familiar environmental sounds (Specht and Reul, 2003; Thierry et al., 2003; Thierry and Price, 2006). Together, these studies are consistent with the previous claims that the posterior superior temporal cortex is related to sound familiarity while the anterior superior temporal gyrus is related to the acoustic complexity of speech.

Critically, speech selective responses (more activated for speech than nonspeech sounds) do not imply “speech specificity” because both the anterior and posterior temporal speech areas each respond to nonspeech stimuli (e.g. environmental sounds, pitch changes, melodies, familiarity or conceptual processes). It therefore became apparent that specialisation for speech is not localized in dedicated brain areas but is characterised by a distributed pattern of activity across many different areas that are each involved in speech and nonspeech processing, depending on the type of task (Price et al., 2005).

In the left inferior frontal areas that were activated during auditory categorisation tasks (Auditory processing that is common to speech and nonspeech sounds section) and phonological discrimination tasks (Homae et al., 2002; Booth et al., 2003; Burton et al., 2005; Husain et al., 2006), there were increased efforts to dissociate different levels of processing such as selecting, segmenting and comparing (Burton and Small, 2006). Consistent with previous reports, (i) posterior inferior frontal activation (BA 44) was associated with articulatory recoding (Burton et al., 2005) or decision mechanisms (Binder et al., 2004); (ii) the left ventral premotor cortex was also associated with articulatory recoding when participants passively listened to unfamiliar speech sounds (Wilson et al., 2004; Wilson and Iacoboni, 2006); (iii) ventro-lateral prefrontal cortex was associated with working memory demands and (iv) the mid-dorsolateral prefrontal cortex was associated with stimulus monitoring (Burton et al., 2005). Altogether, there was a growing perspective that inferior frontal or premotor activations during auditory speech processing were the consequence of top-down supplementary mechanisms that constrain bottom up speech processing in temporal regions (Zekveld et al., 2006), particularly when speech is unfamiliar or ambiguous (Dehaene-Lambertz et al., 2005). In addition, there were reports that the left inferior frontal cortex was activated when observing nonverbal actions with the suggestion that it might be involved in the interpretation of movements in general rather than being specific to articulation (Fadiga et al., 2006).

#### Speech selective auditory responses. Time era: 2007–2011

##### Extending prior findings

As shown previously, activation for auditory speech processing was primarily left lateralized when other factors were controlled (Wolmetz et al., 2011), the speech selective response in the left anterior superior temporal cortex was related to the acoustic content of the stimulus (Leaver and Rauschecker, 2010; Agnew et al., 2011; Leff et al., 2009; Rauschecker and Scott, 2009; Specht et al., 2009; Friederici et al., 2010; Obleser and Kotz, 2010); and the speech selective response in the left posterior superior temporal cortex was related to (a) the familiarity of auditory spectral patterns (Leech et al., 2009; Liebenthal et al., 2010; Margulis et al., 2009; Dick et al., 2011), (b) the maintenance phase of phonological working memory (Strand et al., 2008) and (c) the perception of hearing speech (Heinrich et al., 2008).

Activation in the left premotor cortex during speech processing was associated with better perceptual performance (Callan et al., 2010), particularly when the acoustic input was sparse (Osnes et al., 2011). However, as premotor activation was not found to be sensitive to articulatory complexity during speech perception, Tremblay and Small (2011a) suggested that motor representations were incompletely activated during perception. The premotor response during perception was also found for non‐speech sounds (Agnew et al., 2011). This implies that either the premotor response was not involved in articulation or that subarticulatory activation occurs during the perception of non-speech sounds.

In the left posterior part of the inferior frontal gyrus and inferior parietal regions, activation was associated with processing syllable order (Moser et al., 2009) or discriminating sounds on the basis of subtle temporal acoustic features that are typical of phoneme categories (Zaehle et al., 2008; Raizada and Poldrack, 2007; Turkeltaub and Coslett, 2010). This might be explained by prior association of these areas with auditory short-term memory (Strand et al., 2008). There was continued support for the view that the left posterior inferior frontal activation is related to articulatory processes because it was positively correlated with the phonotactic frequency (the pronounceability of combinations of phonemes) of heard sounds (Vaden et al., 2011). Less support was provided for the prior association of the left ventral anterior supramarginal gyrus with a phonological store because the fronto-parietal verbal short-term memory areas are also activated for unexpected auditory change during tasks that have minimal working memory demands (Myers et al., 2009; Zevin et al., 2010; Ravizza et al., 2011). This suggested that left fronto-parietal responses during speech may be related to nonverbazl auditory attention and categorization functions that provide top-down regulation of auditory functions (Elmer et al., 2011b; Davis et al., 2007; Ravizza et al., 2011; Zevin et al., 2010).

#### Speech selective auditory responses: 20 year summary

Depending on the task, left lateralized activation for speech relative to acoustically matched nonspeech sounds was reported in: (1) the left posterior superior temporal cortex (pSTS in Table 2 and Fig. 3) where it was consistently related to sound familiarity; (2) the left anterior superior temporal gyrus (aSTS in Table 2 and Fig. 3) where it was consistently related to the acoustic complexity of speech; (3) the left inferior frontal and premotor areas (LpOp and vPM in Table 2 and Fig. 3) where it was consistently related to articulatory re-coding that places top-down constraints on the disambiguation of speech sounds; and (4) the ventral supramarginal gyrus (vSMG in Table 2 and Fig. 3) where there is accumulating evidence of involvement in auditory attention and categorization functions that that are not specific to speech.

Overall, the results suggest that phonological processing of speech sounds arises from the functional integration of acoustic processing (in temporal lobe regions) and articulatory processing (in premotor and frontoparietal regions). This is consistent with the growing appreciation that speech processing areas are activated by non-speech stimuli (Zaehle et al., 2008; Schon et al., 2010) and that functional specialisation arises in the network of regions that are activated (Hein and Knight, 2008; Londei et al., 2010). Another important step forward was to consider how different parts of the network parcellated into different processing streams (Hickok and Poeppel, 2007; Rauschecker and Scott, 2009) and how these streams are supported by anatomy (Saur et al., 2008) and functional connectivity (Obleser et al., 2007a; Londei et al., 2010; Leff et al., 2008; Schofield et al., 2009; Osnes et al., 2011; Eickhoff et al., 2009; Nath and Beauchamp, 2011).

### Speech comprehension

Speech comprehension occurs when familiar sounds are recognised and mapped onto their meanings. Many cognitive processes are involved. For example, short-term auditory memory is required because speech is a serial dynamic auditory signal that needs to be integrated over time, particularly when multiple words are presented in a sentence. Accessing meaning also requires selection from multiple competing representations of speech sounds that may sound very similar (sun, some) or the same (sun, son), but have different meanings that are determined by the context. Given the complexity of the processes involved, an early distinction was made between semantic representations and task dependent “strategic/executive/control” processes that are required to access, retrieve, compare and manipulate semantic knowledge.

#### Speech comprehension. Time era: 1992–1996

##### Novel findings

A direct comparison of semantic decisions on auditory words to phonological decisions on nonwords demonstrated widely distributed activation in the left middle and inferior temporal gyri, left superior frontal cortex and bilateral angular gyri (Demonet et al., 1992, 1994a). These early Demonet et al. studies also played an important role in dissociating (a) temporal lobe regions involved in pre-lexical processing (bilateral superior temporal gyri) from temporal lobe regions involved in semantic processing (left middle and inferior temporal gyri); and (b) parietal lobe regions involved in phonological decision strategies (supramarginal gyri) from parietal lobe regions involved in semantic processing (angular gyri). Subsequently, the role of the left middle and inferior temporal gyri and left angular gyrus was also reported for semantic decisions on written words and pictures (Vandenberghe et al., 1996). This highlighted an amodal semantic system that was common to auditory words, written words and (nonverbal) pictures. Together these studies suggested that semantic decisions on the meaning of words involve the left middle and inferior temporal and angular gyri while phonological decisions on the sound content of words involve the supramarginal gyri and the left posterior inferior frontal gyrus.

###### Sentences and narratives

At the narrative level, Mazoyer et al. (1993) demonstrated that listening to meaningful stories, relative to unmeaningful speech, increased activation in bilateral temporal poles and Bottini et al. (1994) demonstrated that comprehension of sentences (compared to a lexical-decision task) induced extensive activation in several regions of the left hemisphere, including the prefrontal and basal frontal cortices, the middle and inferior temporal gyri and temporal pole, the parietal cortex and the precuneus. In addition, when the sentences had metaphorical meanings, there was right hemisphere activation in the prefrontal cortex and middle temporal gyrus and posterior cingulate. Within this widely distributed system, activation could relate to many different levels of processing such as auditory short-term memory, grammatical deconstruction or semantic processing (Stromswold et al., 1996). Although the studies in this era were not able to interpret the contribution of each of the areas reported, the results showed that language comprehension involves widespread distributed systems in both left and right hemispheres. This pushes us way beyond the appealing simplicity of the early neurological model.

#### Speech comprehension. Time era: 1997–2001

##### Extending prior findings

###### The widely distributed semantic system

The involvement of widely distributed temporal, parietal and frontal brain areas in speech comprehension continued to be reported (Chee et al., 1999; Hashimoto et al., 2000; Benson et al., 2001; Newman and Twieg, 2001; Vouloumanos et al., 2001; Binder et al., 1997; Nakai et al., 1999; Newman et al., 2001). These effects are more likely to reflect amodal semantic processing than access to semantics from speech sounds because they were commonly activated irrespective of whether the stimuli were auditory or visual words (Chee et al., 1999). When access to semantic associations was made more challenging, activation in the prefrontal and angular gyri increased: for example, when speech complexity increased (Benson et al., 2001), when reading sentences with semantic violations (Newman et al., 2001) or when listening to a non-native language (Nakai et al., 1999).

###### Sentences and narratives

There was continued emphasis on the importance of Broca's area (BA 44 and sometimes BA 45) for syntactic processing (Caplan et al., 1998,1999; Inui et al., 1998; Kang et al., 1999; Moro et al., 2001; Nakai et al., 1999; Ni et al., 2000) even when articulation was suppressed (Caplan et al., 2000). Although the same frontal lobe regions were activated by semantic processing (Nakai et al., 1999), morphologically complex words (Laine et al., 1999) and lexical decisions on verbs relative to nouns (Perani et al., 1999), a striking double dissociation was observed with greater activation in the left dorsal pars opercularis for syntactic than semantic processing (Kang et al., 1999; Dapretto and Bookheimer, 1999) and greater activation for semantics than syntactics in the ventral inferior frontal gyrus (BA 47, pars orbitalis; Dapretto and Bookheimer, 1999) or the right inferior frontal lobe (Kang et al., 1999). However, other studies did not show such a clear cut dissociation between semantic and syntactic processing (Kuperberg et al., 2000; Tyler et al., 2001).

##### Novel findings

The importance of the auditory processing pathways that projected anteriorly from Heschl's gyrus was highlighted by Scott et al. (2000) who identified a region in the left anterior superior temporal sulcus (aSTS) that was activated by intelligible speech when all other characteristics of speech were controlled. Greater aSTS activation was also reported for words relative to syllables (Giraud and Price, 2001) and auditory sentence processing relative to environmental sounds (Humphries et al., 2001), with activation extending into the left temporal pole for higher-level discourse processing (Maguire et al., 1999). Other areas associated with speech comprehension included the left medial temporal cortex (Newman et al., 2001) and right temporal and frontal regions (Kang et al., 1999; Kuperberg et al., 2000; Newman et al., 2001; Robertson et al., 2000; Meyer et al., 2000), although the contribution of each area remained unclear.

The substantial overlap in the areas involved in speech comprehension as well as production was reported by Wise et al. (2001) and Papathanassiou et al. (2000). For example, Papathanassiou et al. (2000) reported activation common to both listening to stories and verb generation in the pars opercularis and triangularis of the inferior frontal gyrus (Broca's area), the posterior part of the superior temporal cortex centred around the superior temporal sulcus (Wernicke's area) but extending into the posterior part of the planum temporale and the most anterior part of the left inferior temporal gyrus at the junction with the anterior fusiform gyrus (the basal temporal language area) and the inferior and lateral parts of the right cerebellar cortex. This overlap is likely to reflect the many processes that are common to speech production and comprehension, for example, semantic processing, articulatory planning and short term memory. A review of neuroimaging studies of semantic processing in this time era can be found in Bookheimer (2002).

###### Sentences and narratives

New areas associated with syntactic processing during comprehension were the right inferior frontal regions (Friederici et al., 2000; Moro et al., 2001; Kang et al., 1999), the left caudate nucleus (Moro et al., 2001), insula (Moro et al., 2001), planum polare bilaterally (Meyer et al., 2000; Friederici et al., 2000) and the superior frontal cortex (Newman et al., 2001). Reviews of syntactic processing at this time can be found in Caplan (2001) and Sakai et al. (2001).

#### Speech comprehension. Time era: 2002–2006

##### Extending prior findings

###### The widely distributed semantic system

When accessing semantics from speech sounds, activation was reported to extend from the superior temporal gyri ventrally into the left middle temporal cortex (BA 21; Kotz et al., 2002; Booth et al., 2002a; Giraud et al., 2004; Hickok and Poeppel, 2004; Meyer et al., 2005); dorsally and posteriorly into the left posterior temporoparietal cortex (Roder et al., 2002; Hickok and Poeppel, 2004) and anteriorly into the ventral anterior temporal cortex (BA 38; Crinion et al., 2003; Giraud et al., 2004; Humphries et al., 2005; Meyer et al., 2005; Narain et al., 2003; Noppeney and Price, 2002; Roder et al., 2002). When the demands on semantic retrieval increased, activation was also observed in the ventral inferior frontal cortex (Rodd et al., 2005; Noppeney and Price, 2002), the left fusiform gyrus (Adams and Janata, 2002), and the angular gyri (Homae et al., 2002; Noppeney and Price, 2002; Schmithorst et al., 2006). All these areas were commonly activated during comprehension of spoken and written language (Spitsyna et al., 2006). They are therefore not specific for accessing semantics from auditory speech.

###### Dissociating the roles of different semantic areas

Within the amodal semantic system, the left anterior temporal pole was particularly involved in specific semantic representations (Bright et al., 2004) and for meaningful relative to meaningless sentences (Vandenberghe et al., 2002; Roder et al., 2002; Xue et al., 2005). The ventral inferior frontal cortex was associated with strategic semantic processing (Adams and Janata, 2002; Booth et al., 2002a; Crinion et al., 2003; Homae et al., 2002; Noppeney and Price, 2002; Noesselt et al., 2003; Badre et al., 2005), more dorsal posterior frontal areas were associated with attention demanding speech comprehension tasks (Giraud et al., 2004; Adams and Janata, 2002; Noesselt et al., 2003) and the left superior frontal gyrus was activated when a word's meaning depended on semantic context (Scott et al., 2003).

###### Sentences and narratives

The influence of grammatical analysis or morpho-phonological segmentation on inferior frontal activation continued to be emphasized (Sakai et al., 2003; Suzuki and Sakai, 2003; Tyler et al., 2005), with claims that the left prefrontal cortex is more specialised for syntactic processing during sentence comprehension than other domain-general processes such as short-term memory (Sakai et al., 2003). Likewise, the role of the basal ganglia in syntactic processing continued to be of interest (Friederici et al., 2003) with Friederici and Kotz (2003) proposing that, while inferior frontal and anterior temporal cortices were involved in early syntactic processing, the basal ganglia were involved in late syntactic processing. Other studies emphasized that sentence comprehension is a complex task that involves both language-specific processing components and general cognitive resources that recruit the anterior cingulate, premotor cortex and prefrontal lobe regions regardless of syntactic complexity (Peelle et al., 2004).

#### Speech comprehension. Time era: 2007–2011

##### Extending prior findings

###### The widely distributed semantic system

As shown previously, increasing attention to the meaning of speech involves left lateralized activation extending anteriorly, laterally, ventrally and posteriorly from Heschl's gyri in multiple different pathways (Sharp et al., 2010; Davis and Gaskell, 2009; Kotz et al., 2010; Kouider et al., 2010; Obleser et al., 2007b; Devauchelle et al., 2009; Obleser and Kotz, 2010; Hubbard et al., 2009; Kircher et al., 2009b; Mashal et al., 2009; Rogalsky and Hickok, 2009; Visser et al., 2010; Tyler et al., 2010; Ye and Zhou, 2009). In the temporal lobe, activation extended into the anterior and posterior areas in the middle temporal gyrus, posterior inferior temporal gyrus, anterior fusiform and the hippocampus (in medial temporal cortex). In the parietal lobe, semantic activation was reported in the posterior temporoparietal cortex, the left angular gyrus and precuneus. In the frontal lobe, semantic activation was reported in the left pars orbitalis and middle and superior frontal gyri. These areas were again reported for semantic processing of written words and pictures of objects (Binder et al., 2009; Diaz and McCarthy, 2009), consistent with their role in amodal conceptual processing or semantic control (Visser et al., 2010; Visser and Lambon Ralph, 2011; Rogalsky and Hickok, 2009; Snijders et al., 2009; Richardson et al., 2010).

The emphasis on left lateralized activation does not exclude the contribution of the right hemisphere homologue areas which were shown to be particularly involved during tasks that required executive processing (Vigneau et al., 2011) and/or the integration or consolidation of semantic concepts, for example, when the words “boat” and “house” occur together, they refer to a single concept meaning “a shelter for boats” (Graves et al., 2010b).

###### Dissociating the roles of different semantic areas

The functional role of each semantic area unravelled further, with the involvement of each area depending on the task demands and the baseline condition. Anterior temporal activation was linked to semantic associations because it was observed during passive listening (Jobard et al., 2007; Awad et al., 2007), except when the baseline task was at rest which was argued to inadvertently control for semantic processing because free flowing thought is richly semantic in nature (Binder et al., 2011). Posterior middle temporal activation was more sensitive to semantic demands because, although it was observed during passive listening (Jobard et al., 2007; Awad et al., 2007), activation increased with executive demands (Whitney et al., 2011) and when semantic information was received in both auditory and visual modalities simultaneously (Holle et al., 2010; Kircher et al., 2009a, 2009b; Robins et al., 2009; Dick et al., 2009). Activation in ventral frontal regions in the left pars triangularis/orbitalis also increased with the executive demands of the task (Whitney et al., 2011), for example during conditions where competition from semantically similar targets was high versus low (Zhuang et al., 2011). This is consistent with a role in selection and retrieval of semantic representations. Parietal activation in the angular gyrus and retrosplenial/posterior cingulate was not typically observed during passive listening but was observed during lexical decisions on words compared to pseudowords (Raettig and Kotz, 2008), during memory demanding comprehension of narratives (Awad et al., 2007). The left medial parietal cortices (precuneus and posterior cingulate) were associated with updating story representations in narrative language comprehension (Whitney et al., 2009a). In contrast, the angular gyri were associated with integrating and retrieving concepts (Binder et al., 2009) and top-down predictions of semantic content (Obleser et al., 2007b; Obleser and Kotz, 2010; Carreiras et al., 2009; Brownsett and Wise, 2010; Sharp et al., 2010). It also became apparent that there were several different functionally distinct subregions in the angular gyrus (Seghier et al., 2010). For more comprehensive reviews of the semantic system, see Binder et al. (2009) and Binder and Desai (2011).

###### Sentences and narratives

Dorsal frontal regions in the left premotor cortex and pars opercularis were more activated when sentence meaning was ambiguous or implausible relative to plausible (Tyler et al., 2010; Obleser and Kotz, 2010; Desai et al., 2010; Turner et al., 2009; Szycik et al., 2009; Ye et al., 2009; Bilenko et al., 2009; Mashal et al., 2009; Willems et al., 2009; Desai et al., 2010). Explanations for why activation in these areas is greater for implausible sentences include the following: (a) activation in the left premotor cortex reflects covert articulatory processing (Rogalsky et al., 2008; Iacoboni, 2008; Callan et al., 2010; Osnes et al., 2011; Adank and Devlin, 2010; Meister et al., 2007; Scott et al., 2009a; Hickok, 2009; Hickok et al., 2011); (b) activation in the ventral pars operculum reflects articulatory planning (Papoutsi et al., 2009) and verbal working memory (Koelsch et al., 2009); (c) activation in the left dorsal pars opercularis reflects linguistic and non-linguistic sequencing (Price, 2010), decision making (Heim et al., 2007) and top-down processing that attempts to predict the most likely meaning or sound (Obleser et al., 2007a; Davis and Johnsrude, 2007) while (d) activation in the right pars opercularis reflects conflicting semantic information (Snijders et al., 2009; Dick et al., 2009; Zhuang et al., 2011) and nonlinguistic executive and attentional processing (Vigneau et al., 2011). A 20 year review of semantics and syntax can be found in Cappa (2011).

#### Speech comprehension: 20 year summary

Many left lateralized areas are involved in accessing semantics from auditory speech and other stimuli. Anterior temporal areas (aSTS, LaMTG/LaITG in Table 2 and Fig. 3) are involved in accessing increasingly specific semantic associations, with activation for sentences and narratives spreading anteriorly into the temporal pole. Posterior temporal areas (pMTG, pITG in Table 2 and Fig. 3) are increasingly involved as the semantic content or task demands increase. Ventral inferior frontal areas (pOrb and pTri in Table 2 and Fig. 3) are involved in selecting and retrieving task related semantic attributes. The dorsal superior frontal gyrus (SFG in Table 2 and Fig. 3) is also involved, albeit less consistently, in constraining semantic processing. The angular gyri (ANG in Table 2 and Fig. 3) have been associated with the crossmodal integration of semantic features and in predicting the semantic nature of the stimulus while more medial parietal areas (precuneus and posterior cingulate) are more involved for narrative than single word comprehension. The above descriptions of the functional role of each region are clearly vague and insufficient. The anatomical localization of the anatomical regions is also insufficiently precise. We are therefore far from understanding how the human brain supports speech comprehension.

### Speech production

The processing involved in speech production overlaps with that involved in speech comprehension (Papathanassiou et al., 2000). For example, both involve access to the semantic system. In addition, subarticulatory processing may be automatically involved in discriminating auditory words while auditory imagery may be automatically involved in articulation. This section focuses on those processes that are more involved in production than comprehension. There are no separate sections for word and sentence production because these have been shown to involve the same neural structures (Tremblay and Small, 2011b).

#### Word retrieval from semantics. Time era: 1992–1996

##### Novel findings

The left prefrontal activation associated with word generation included dorsal and ventral inferior frontal areas (BA 44, BA 45, BA 47) and medial frontal areas (McCarthy et al., 1993; Raichle et al., 1994; Buckner et al., 1995). The middle frontal cortex (BA 46) and medial frontopolar prefrontal cortex (BA 10) were related to semantically driven word retrieval because they were more activated during verb generation (generate “eat” in response to “cake”) relative to phonologically constrained stem completion (generate “green” in response to “GRE”). In contrast, the posterior inferior frontal regions (near BA 44 or BA 45) were activated during stem completion as well as verb generation (Buckner et al., 1995) suggesting a less semantically determined role in word retrieval. With practice, activation in all these prefrontal areas decreased while it increased in the bilateral sylvian-insular cortex, as predicted by prior claims that the insula was more involved in automatic speech production than word retrieval (Mohr, 1978).

The temporal lobe areas associated with word retrieval tasks included: the left temporoparietal cortex, left posterior superior temporal gyrus, left posterior inferolateral temporal cortex and bilateral superior temporal gyri (Fiez et al., 1996a; Warburton et al., 1996; McGuire et al., 1996a; Paus et al., 1996a, 1996b; Price et al., 1996b, 1996c). The left posterior temporoparietal cortex and the left inferolateral temporal cortex were more involved in producing words than listening to words (Warburton et al., 1996), the left posterior superior temporal gyrus was commonly activated during speech production and perception (Howard et al., 1992; Warburton et al., 1996; Fiez et al., 1996a) and activation in the bilateral superior temporal gyri during overt speech production was attributed to auditory processing of the participants ‘own voice’ because the same regions were also activated when listening to ‘another's voice' from a pre-recorded tape (Price et al., 1996a; McGuire, 1996b), see Auditory‐motor feedback during speech production section below.

#### Word retrieval from semantics. Time era: 1997–2001

##### Extending prior findings

The left anterior frontal areas (middle frontal/BA 46 and pars triangularis/BA 45) were consistently reported during the most demanding word retrieval tasks such as verbal fluency (Paulesu et al., 1997), sentence generation (Muller et al., 1997; Kircher et al., 2001) and some picture naming paradigms (Murtha et al., 1999), but not other plausibly easier picture naming tasks (Etard et al., 2000). Functionally, these prefrontal activations were associated with: (i) semantic working memory regardless of stimulus modality (Gabrieli et al., 1998; de Zubicaray et al., 1998; Smith et al., 1998), (ii) the selection of words from many competing alternatives (Thompson-Schill et al., 1999), and (iii) response suppression (De Zubicaray et al., 2000; Collette et al., 2001).

##### Novel findings

The anterior cingulate was also associated with response suppression during verbal fluency tasks (Barch et al., 2000; De Zubicaray et al., 2000; Lurito et al., 2000). For example, Barch et al. (2000) found that the anterior cingulate cortex was activated during a verb-generation task when there was competition among alternative responses. This functional account of anterior cingulate activation explains the higher response in this area during sentence completion than reading aloud (Kircher et al., 2001) because multiple meanings are generated in the course of producing an appropriate completion. Crosson et al. (1999) also showed that the part of the anterior cingulate that was relevant to word generation was a posterior dorsal subregion that is anatomically connected to lateral motor systems.

In the left posterior inferior temporal lobe, activation was again reported during semantically mediated word retrieval tasks such as picture naming (Zelkowicz et al., 1998; Moore and Price, 1999) and sentence generation (Muller et al., 1997) but the more medial aspect of this area, in the vicinity of the occipitotemporal sulcus, was also observed for the retrieval of colour and letter names that place minimal demands on semantics (Price and Friston, 1997). The association of this posterior inferior temporal region with word retrieval in the absence of visual or auditory stimuli was unexpected (Braun et al., 2001) but consistent with observations that damage to the posterior inferior temporal cortex causes anomia (Hillis et al., 2005) and electrical stimulation to the inferior temporal cortex caused receptive and expressive language difficulties (Luders et al., 1991). It was nevertheless clear that there are multiple posterior inferior temporal areas involved in word retrieval (Murtha et al., 1999; Moore and Price, 1999) including: (i) a lateral posterior inferior temporal region involved in generating verbs and nouns from heard words (Warburton et al., 1996); (ii) the anterior fusiform gyrus that was commonly activated for word retrieval and comprehension (Papathanassiou et al., 2000) but more activated for semantic than phonological decisions (Mummery et al., 1998; Chee et al., 1999); and (iii) more posterior inferior temporal/occipitotemporal regions that are more involved in naming than viewing pictures, words, letters or colours (Price and Friston, 1997).

In the left posterior superior temporal cortex, Burton et al. (2001) and Giraud and Price (2001) replicated the Howard et al. (1992) study by showing activation for word repetition when sensorimotor activation is controlled. Wise et al. (2001) advanced this finding by showing that it was the most posterior and medial part of the planum temporale, at the junction with the inferior parietal lobe (i.e. the temporo-parietal junction), which was more activated by speech production than speech perception, even though this region was consistently associated with auditory speech processing that did not involve speech production (See Auditory processing that is common to speech and nonspeech sounds and Speech selective auditory responses).

In an extended review of studies published up until 2001, Indefrey and Levelt (2004) associated: (a) the mid part of the left middle temporal gyrus with semantically driven selection of a lexical item because it was commonly activated by word generation and picture naming but not by word reading; (b) the posterior middle and superior temporal gyri with lexical word form retrieval from a phonological store because it was commonly activated by picture naming, word generation, and word reading, but not pseudoword reading and (c) frontal lobe areas with later stages of speech production such as articulatory planning (see Covert articulatory planning section for rationale).

#### Word retrieval from semantics. Time era: 2002–2006

##### Extending prior findings

There was a relative paucity of speech production studies published in this time era because fMRI scanners had largely replaced PET scanners and investigators were concerned about the potential interference from head motion on BOLD signal during speech production (Gracco et al., 2005; Lurito et al., 2000). Nevertheless, the brain areas associated with word retrieval in previous years were largely replicated with refined function and further anatomical dissociations (Kircher et al., 2004; de Zubicaray et al., 2006; Kemeny et al., 2006; Sharp et al., 2005). This resulted in a clearer appreciation that word retrieval involved: (a) the anterior rather than the posterior left prefrontal cortex (BA 45/46 versus BA 44; Amunts et al., 2004; Tremblay and Gracco, 2006; Haller et al., 2005); and (b) lobules VI and VIIB in the right posterior lateral cerebellum in those with left lateralized frontal activation (Jansen et al., 2005; Frings et al., 2006) or the left homologues of these areas in those with right lateralized frontal activation (Jansen et al., 2005).

##### Novel findings

In the right pars opercularis (BA 44), activation was found to be reduced for the generation of narrative (propositional) speech compared to a baseline nonspeech condition (Blank et al., 2003) with no right frontal activation for generating words relative to generating nonsense syllables (Crosson et al., 2003). Crosson et al. (2003) hypothesized that the right basal ganglia (caudate nucleus and putamen) suppressed right frontal activation to reduce interference during language and this resulted in left lateralized activation in the inferior frontal, pre-SMA, thalamus and basal ganglia regions. However, others found that right prefrontal activation increased when word selection became more difficult, for example when the constraints on word selection were reduced (Vartanian and Goel, 2005) or during the generation of narratives (Howard-Jones et al., 2005). As there are several different frontal regions being referred to, in two different hemispheres, and under various tasks, the determinants of right frontal activation remained unclear.

#### Word retrieval from semantics. Time era: 2007–2011

##### Extending prior findings

As shown previously, word retrieval was strongly associated with activation in the left anterior prefrontal cortex including BA 45 in the left inferior frontal cortex and BA 46 in the left middle frontal gyrus, even when articulation and conceptual processing were controlled (Spalek and Thompson-Schill, 2008; Whitney et al., 2009b; Jeon et al., 2009; Heim et al., 2009a; de Zubicaray and McMahon, 2009) and this was accompanied by activation in the right vermal and hemispheric components of lobule VI and Crus I and II of lobule VII in the posterior lateral cerebellum (Krienen and Buckner, 2009; Murdoch, 2010; Stoodley and Shamahmann, 2009, 2010). Right frontal activation was more likely to be observed in the context of more retrieval effort (Sachs et al., 2011) which may explain the corresponding increases during paced relative to unpaced word production (Basho et al., 2007) and for older relative to younger participants (Meinzer et al., 2009). Within the anterior cingulate, an anterior zone was associated with conflict monitoring (response suppression) which might be more related to word retrieval than the posterior zone associated with general response selection (Schulze et al., 2011).

##### Novel findings

When word retrieval became more semantically demanding, for example when subjects were instructed to retrieve narratives or when distracting semantic information was added, activation for word retrieval was also reported in the medial superior frontal cortex (Birn et al., 2010), the left pars orbitalis in the ventral inferior frontal cortex (de Zubicaray and McMahon, 2009; Saur et al., 2008), the left angular gyrus/inferior parietal cortex (Mechelli et al., 2007; Troiani et al., 2008; Ye et al., 2011), left ventrolateral temporal cortex (Dhanjal et al., 2008; Spalek and Thompson-Schill, 2008) and the left hippocampus (Hocking et al., 2009; Whitney et al., 2009b). However, these anatomically distributed brain areas are likely to reflect silent conceptual processing (see Speech comprehension section) rather than word retrieval per se.

When word retrieval became more demanding, in the context of minimal semantic constraints, activation was reported at the left temporoparietal junction, for example, when producing picture names with low relative to high word frequency (Graves et al., 2007; Wilson et al., 2009), for words which lack a phonological neighbour (Peramunage et al., 2011) and in the presence of phonological relative to semantic interference (Abel et al., 2009; Peschke et al., 2012). As the same temporoparietal area was activated when the post-articulatory auditory feedback was altered to create a conflict between the expected auditory response to the spoken name and the actual auditory response (Zheng et al., 2010), the increased temporoparietal activation during challenging word retrieval tasks may simply be a consequence of increased verbal self-monitoring (Hocking et al., 2009; Price, 2010) during which the word produced is compared with the intended output; see Auditory‐motor feedback during speech production section on post articulatory auditory motor feedback below for further discussion.

#### Word retrieval from semantics: 20 year summary

The most consistent activation for word retrieval from semantics when articulation and semantics are controlled were: the left middle frontal gyrus (MFG in Table 2 and Fig. 3), Crux 1 of the right lateral (l) posterior (p) cerebellum (CB) and posterior regions in the left middle and inferior temporal cortices (L.pMTG and LpITG in Table 2 and Fig. 3), with more dorsal superior temporal lobe regions (pSTG and TP in Table 2 and Fig. 3) when retrieval becomes more difficult and an anterior zone in the anterior cingulate (ACC a zone in Table 2 and Fig. 3) associated with response suppression.

### Covert articulatory planning

Premotor stages of speech production can be investigated with covert articulation tasks that involve the silent production of speech sounds without movements in the articulators or phonation. In addition to motor planning, this level of processing can evoke “inner speech” because the auditory images of speech can be heard in our heads when we prepare to articulate words even though there is no auditory stimulation.

#### Covert articulatory planning. Time era: 1992–1996

##### Novel findings

During silent articulation (McGuire et al., 1996a,b) and verbal working memory (Paulesu et al., 1993; Fiez et al., 1996b; Salmon et al., 1996; Schumacher et al., 1996), activation in the left posterior inferior frontal gyrus (Broca's area, BA 44) and/or left premotor cortex (BA 6) was associated with “inner speech”. This was consistent with the view that the left posterior inferior frontal gyrus was involved in auditory-articulatory mapping which resulted in subvocal articulation during perception and auditory imagery of speech during articulation.

#### Covert articulatory planning. Time era: 1997–2001

##### Extending previous findings

Covert articulation was again associated with activation in the left posterior inferior frontal region (pars opercularis, BA 44) because this area was activated during: silent word generation (Papathanassiou et al., 2000), subvocal rehearsal of phonological information (Smith et al., 1998), silent syllable counting when presented with written pseudowords (Poldrack et al., 1999), segmentation of speech sounds (Burton et al., 2000) and by letter relative to semantic fluency (Paulesu et al., 1997). There were two unexpected observations. The first was that there was surprisingly little posterior inferior frontal activation during auditory word repetition and reading aloud (Karbe et al., 1998; Wise et al., 1999) with no significant activation difference between word or pseudoword repetition (Newman and Twieg, 2001) but consistently higher activation for reading pseudowords than words (Fiez et al., 1999; Bokde et al., 2001). This suggests that left posterior inferior frontal activation was less in the context of phonological cues and more when the articulatory associations of the word were unknown or needed to be held in short term memory. The second unexpected observation was that it became increasingly apparent that activation in Broca's area was not specific to speech. For example, Binkofski et al. (2000) reported left lateralized posterior inferior frontal activation for imagining a moving target and suggested that it was involved in mediating higher-order movement control of forelimbs as well as speech. This observation indicates that the left posterior inferior frontal cortex is more involved in motor planning than in auditory imagery or inner speech.

##### Novel findings

The contribution of the left anterior insula to articulatory planning was suggested in both functional imaging (Wise et al., 1999) and lesion (Dronkers, 1996) studies. This explained why the left anterior insula was activated during a range of tasks including overt picture naming (Price et al., 1996b; Zelkowicz et al., 1998; Etard et al., 2000). Greater left anterior insula activation for overt than covert articulation also suggested that the left insula might be more directly involved in the coordination of the various (up to 100) muscles engaged in articulation and phonation (Riecker et al., 2000).

Activation in the SMA and bilateral anterior cerebellum (lobules IV and V) during silent articulation tasks (Kawashima et al., 2000) suggested that the contribution of these regions to other tasks such as overt picture naming (Etard et al., 2000), vocalisation (Hirano et al., 1997) and breathing (Murphy et al., 1997) occurred prior to vocalisation. A role for the SMA in the timing of speech and nonspeech motor commands was suggested following observations that the SMA was activated during silent articulation and memory-timed finger movements (Kawashima et al., 2000). In contrast, Ackermann et al. (1998) proposed that cerebellar activation during silent articulation was the consequence of subliminal activity of orofacial and laryngeal muscles.

#### Covert articulatory planning. Time era: 2002–2006

##### Extending previous findings

The opercular part of the posterior inferior frontal cortex (pars opercularis, Broca's area, BA 44) was associated with forming or dividing words into syllables (syllabification) during both covert (silent speech) and overt (speaking aloud) production (Indefrey and Levelt, 2004; Callan et al., 2006) or translating speech into articulatory code (Hickok and Poeppel, 2004). Within the pars opercularis, a distinction was made between dorsal and ventral parts with the dorsal part activated by action observation as well as imitation but the ventral part activated by imitation more than observation (Molnar-Szakacs et al., 2005). This suggests that the ventral pars opercularis has a more motoric function than the dorsal pars opercularis.

More posteriorly, in the left premotor cortex (BA 6), activation was associated with compiling motor codes for syllables (Indefrey and Levelt, 2004) and a “speech sound map” that linked phonemes or frequent syllables to their associated motor programs (Guenther et al., 2006). Although different parts of the dorsal pars opercularis and premotor cortex were found to be responsive to the observation of hand, mouth and foot movements (Binkofski and Buccino, 2006), no speech specific areas were identified when speech was compared to nonspeech motor functions (Gelfand and Bookheimer, 2003; Brown et al., 2006). For example, the dorsal pars opercularis was associated with sequencing the motor acts required to produce hummed notes as well syllables (Gelfand and Bookheimer, 2003).

##### Novel findings

A rostrocaudal gradient distinction was made between the pre-SMA and the more posterior SMA-proper: During silent articulation, the pre-SMA was associated with sequencing abstract motor plans while the SMA-proper was associated with the control of motor output (Alario et al., 2006). This distinction was supported by observations that the SMA was commonly activated by word generation and reading when articulation was matched but the pre-SMA was more activated by: (a) word generation than reading, (Chung et al., 2005; Tremblay and Gracco, 2006;), (b) during the covert rather than overt stage of object and action naming (Kemeny et al., 2006), and (c) pseudoword repetition when the phonological complexity of the stimulus increased (Bohland and Guenther, 2006).

#### Covert articulatory planning. Time era: 2007–2011

##### Extending previous findings

In the left pars opercularis (Broca's area/BA 44), a clearer distinction emerged to dissociate the function of the ventral and dorsal parts. The ventral left pars opercularis was activated during covert articulation (Zheng et al., 2010; Papoutsi et al., 2009) with increased activation for phonological relative to semantic retrieval (Heim et al., 2009a), consistent with a role in articulatory recoding of speech (Papoutsi et al., 2009). The left dorsal pars opercularis was associated with processing prior to articulatory recoding (Papoutsi et al., 2009) and was found to be commonly activated by both the production and observation of nonsemantic, nonsyntactic, and nonsense syllables (Fridriksson et al., 2009). The dorsal premotor cortex was associated with general action selection because it was observed during finger movements as well as speech (Meister et al., 2009) while the left ventral premotor cortex was associated with generating the motor acts related to speech sounds (Ghosh et al., 2008; Peeva et al., 2010), nonspeech sounds (Chang et al., 2009) and orofacial movements such as tongue protrusion and lip pursing that are not associated with sound production (Price et al., 2011).

In the pre-SMA, activation was associated with inhibiting rather than initiating vocal and manual responses (Xue et al., 2008). The inhibitory role for the pre-SMA may explain why activation was found to be greater for volitional relative to stimulus driven mouth movements (Tremblay and Gracco, 2010) because selecting the correct response (in volitional mouth movements) requires competing responses to be inhibited. It may also explain the numerous reports that pre-SMA activation is greater for the production of unfamiliar speech sounds (Ghosh et al., 2008; Peeva et al., 2010) in terms of the increased effort in suppressing prepotent familiar speech.

Finally, evidence that activation in the anterior insula, subcortical structures (basal ganglia and thalamus), cerebellum, planum temporale and inferior parietal lobe was reduced during the silent short-term maintenance of auditory stimuli when articulation is suppressed (Koelsch et al., 2009), suggests that these areas are more involved in articulatory activity. Conversely, it was also noted that activation in the planum temporale and inferior parietal lobe was higher during covert than overt production of sentences (Andreatta et al., 2010) and during covert (imagined) than overt singing (Kleber et al., 2007). This suggests that activation in these temporo-parietal regions might be related to the sensorimotor circuits that maintain sound representations for the production of speech and song (Koelsch et al., 2009).

#### Covert articulatory planning: 20 year summary

Covert articulation is a mix of processing that occurs prior to overt articulation and independently from word retrieval. The mapping of heard or intended speech to orofacial movements has been associated with activation in the ventral pars opercularis (pOpv in Table 2 and Fig. 3) and the ventral premotor cortex (vPM in Table 2 and Fig. 3) with subliminal motor activity in lobules IV and V of the bilateral anterior medial cerebellum (a CB). In contrast, mapping in the reverse direction (from orofacial movements to auditory imagery) involves the auditory imagery areas discussed in Auditory processing that is common to speech and nonspeech sounds section (i.e. PT and vSMG in Table 2 and Fig. 3). Covert articulation also activates areas that are engaged by other motor modalities (e.g. fingers), for example, the left dorsal pars opercularis (LpOp-d in Table 2 and Fig. 3) associated with motor sequencing; and the bilateral premotor cortex (d-PreC in Fig. 3).

### Overt articulation during speech production

This section considers the brain areas that control mouth movements (lips, tongue, jaw), the vocal tract (larynx) and breathing during overt speech production.

#### Overt articulation. Time era: 1992–1996

##### Extending previous findings

When syllables were articulated without using the larynx, activation increased in the left primary motor cortex that controls the face, the upper pons, the left planum temporale and the left posterior perisylvian cortex (Paus et al., 1996b). The response in auditory regions (left planum temporale and left posterior perisylvian cortex) was observed even when auditory processing of the spoken response had been minimised and masked out using low-intensity white noise. This was explained in terms of motor activity (left motor primary cortex) causing a discharge corollary of the motor command to sensory structures (Paus et al., 1996b).

##### Novel findings

Tongue movements were found to produce symmetrical activation at the lower primary motor cortex, with left lateralization in the same region during automatic speech and right-sided activation during singing (Wildgruber et al., 1996). There was, nevertheless, a striking overlap between the areas activated during the articulation of speech, vocalisation (Hirano et al., 1996) and the control of volitional breathing in the absence of vocalisation (Ramsay et al., 1993; Fink et al., 1996). For example, controlled breathing activated dorsal primary motor cortices bilaterally, the lateral pre-motor cortex in the right hemisphere, the SMA and left ventrolateral thalamus, with additional activation for expiration in more bilateral ventrolateral primary motor areas. This emphasizes that the motor and premotor activation during speech involves far more than simply moving the mouth.

Investigation of the role of the anterior cingulate cortex in higher-order motor control showed that speech activated the intermediate dorsal and the rostral ACC which is distinct from more anterior regions involved in the control of manual tasks (Paus et al., 1993). The authors proposed that the anterior cingulate participates in motor control by facilitating the execution of the appropriate responses and/or suppressing the execution of the inappropriate ones.

The role of the left putamen in speech production was also discussed in a paper by Klein et al. (1994) who observed that left putamen activation was higher for auditory repetition of words in a non-native language than the native language which can be explained in terms of increased demands on the articulatory system.

#### Overt articulation. Time era: 1997–2001

##### Novel findings

The control of the tongue was localized in the central sulcus (BA 3/4) at approximately 28 mm above the intercommissural plane (Pardo et al., 1997). Contrary to previous findings, activation was observed bilaterally rather than being left lateralized. Corfield et al. (1999) also identified bilateral premotor areas during tongue movements and Lotze et al. (2000) segregated these effects from the primary motor and sensory cortex activations for lip movements. Of more relevance for speech, Lotze et al. (2000) found that the activation for articulating the syllables “pa” and “ta” corresponded to activation related to nonspeech lip and tongue movements respectively.

The control of breathing was investigated by Murphy et al. (1997) who found bilateral sensorimotor and motor cortex activation that was medial to that associated with the articulators. The same study showed that the thalamus was activated during the control of breathing as well as articulation (Murphy et al., 1997) and this may explain some of the left lateralized thalamic activation observed during verbal fluency (Paulesu et al., 1997), naming objects, naming letters, naming colours and reading (Price and Friston, 1997) especially when the rate of speech production increased (Palmer et al., 2001). Activation was also left lateralized in the putamen during an overt versus silent stem completion task (Rosen et al., 2000) and in the posterior pallidum during auditory repetition (Wise et al., 1999). However, while activation in the motor cortex and left thalamus increased with the rate of speech production, activation in the left putamen was higher for slower production rates (Wildgruber et al., 2001). These findings emphasized the importance of left lateralized subcortical responses during speech production and dissociated the function of the left thalamus (associated with breathing) from that in the basal ganglia (associated with the timing of production).

#### Overt articulation. Time era: 2002–2006

##### Extending previous findings

A direct comparison of overt speech production with motor preparation activated regions in the primary motor and somatosensory cortices, SMA, insula, thalamus, basal ganglia and posterior cerebellum (Bohland and Guenther, 2006). The association of the left insula with motor processing rather than articulatory planning was emphasized again following observations that left anterior insula activation was higher for overt than covert speech (Ackermann and Riecker, 2004; Shuster and Lemieux, 2005) and unaffected by the demands on motor planning (Shuster and Lemieux, 2005; Blank et al., 2002). Left lateralized activation during overt articulation was observed in the insula and the dorsolateral premotor cortex and this was contrasted to the bilateral activation in sensorimotor cortex (Riecker et al., 2005). Similarly, left lateralized activation in the primary motor cortex for phonation was contrasted to bilateral activation for tongue movements (Terumitsu et al., 2006).

The motor function of the SMA-proper was re-iterated (Chung et al., 2005; Tremblay and Gracco, 2006; Alario et al., 2006) and associated with the voluntary control of learnt motor sequences of both speech and finger movements (Ullen et al., 2005). This is not incompatible with the involvement of the SMA in the motor control of breathing (see Overt articulation Time era: 1992‐1996 section) which needs to be finely timed with the mouth movements producing sounds. In the cerebellum, the areas activated by speech articulation were in the left and right medial superior posterior cerebellum (paravermal lobule VI) and these areas were separated from the right lateral superior posterior cerebellum (HVI/Crus I) associated with word generation (Frings et al., 2006) and the right inferior posterior cerebellum (HVIIIA) that was activated by vocalisation and breathing during articulation (Nota and Honda, 2004) and during passive listening to auditory clicks that varied in frequency (Ackermann et al., 2001). A distinction was also made between activation for articulation in the left and right medial posterior cerebellum and the striatum (caudate and putamen) because increased rate of articulation had a positive influence on activation in the cerebellar regions and thalamus but a negative influence on activation in the striatum (Riecker et al., 2005, 2006). Thus, the putamen and caudate were more activated for slower (more controlled) speech production. This might explain why left putamen activation was associated with counting (Hinton et al., 2004) and reading written syllables (Bohland and Guenther, 2006) and is consistent with prior claims that the putamen is involved in the timing of speech production.

##### Novel findings

All the above areas (left anterior insula, bilateral premotor and sensorimotor cortices, posterior cerebellum, SMA, thalamus and striatum) were activated for producing melodies (Brown et al., 2006) and whistling (Dresel et al., 2005) as well as speech. This is consistent with specialisation for speech production emerging from the co-ordination of the language system (semantic processing, word retrieval and the sequencing of this information) with mouth movements, vocal tract movements and breathing.

#### Overt articulation. Time era: 2007–2011

##### Extending previous findings

As shown previously, the areas that are activated by speech were also activated by nonspeech orofacial movements and vocal tract gestures (Chang et al., 2009), sniffing (Koritnik et al., 2009), singing (Zarate et al., 2010), volitional exhalation and phonation (Loucks et al., 2007). The contribution of phonation to activation in the bilateral sensorimotor cortex during articulation was also emphasized in several studies (Loucks et al., 2007; Brown et al., 2008, 2009; Grabski et al., 2011; Simonyan et al., 2009; Simonyan and Horwitz, 2011). Brown et al. (2008) identified a larynx-specific region in the motor cortex by comparing vocal and nonvocal laryngeal tasks (phonation) relative to vowel, lip movement, and tongue movement. Grabski et al. (2011) investigated this further describing a dorso-ventral somatotopic organization of lip, jaw, vocal/laryngeal, and tongue movements.

Activation in the left anterior insula, on the junction of the frontal operculum, was sensitive to the complexity or novelty of subsyllabic verbal utterances (Shuster, 2009; Riecker et al., 2008; Moser et al., 2009). Opinions on the role of the insula during articulation changed again with a new focus on its role in the voluntary control of breathing (Ackermann and Riecker, 2010). This is consistent with observations that bilateral insula regions are involved in phonation for speech, volitional exhalation (Loucks et al., 2007) and syllable singing (Brown et al., 2009; Zarate et al., 2010). A role for the insula in the control of breathing may explain why this area is activated during non-verbal orofacial functions including lip movement, tongue movement and vocalisation (Brown et al., 2009) because mouth movements interfere with the regular pattern of breathing thereby increasing the demands on the control of breathing. A similar explanation may account for why bilateral insula activation increases during overt picture naming when phonological/articulatory interference increases (Mechelli et al., 2007). However, it is more difficult to explain how the control of breathing explains bilateral insula activation during silent tasks such as silent rehearsal of tone (pitch) and verbal information (Koelsch et al., 2009) unless breathing is automatically adapted during subvocal articulation.

In the SMA-proper, activation was greater for complex articulation than prolonged vowel production or exhalation (Loucks et al., 2007) and maintained during production consistent with a role in execution as well as initiation (Brendel et al., 2010). In the anterior cingulate, the most posterior zone was associated with motor execution, rather than conflict monitoring or response selection (Schulze et al., 2011) but the anterior zone associated with conflict monitoring (Schulze et al., 2011) was found to be more activated by speech than nonspeech (Chang et al., 2009). Other studies also found the anterior cingulate cortex involved in the suppression of inappropriate and unintended speech (Christoffels et al., 2007; Basho et al., 2007; Ali et al., 2010; Schulze et al., 2011). Such suppression may be less involved in producing nonspeech sounds because selection and production of nonspeech may be slower with less competition from highly similar motor programs.

In the bilateral medial superior posterior cerebellum, activation related to articulation was located in lobule VI/Crus I (Stoodley and Schmahmann, 2009, 2010; Peeva et al., 2010; Durisko and Fiez, 2010). The cerebellum is thought to have a modulatory role in motor functions (Murdoch et al., 2010) and, during articulation, activity in bilateral superior cerebellar regions may contribute to the timing of consonant–vowel syllable production (Ghosh et al., 2008) and the online sequencing of syllables into fast, smooth and rhythmically organized larger utterances (Ackermann, 2008). Lobule VI is associated with lip and tongue movements, therefore Callan et al. (2007) have proposed that it is involved in instantiating internal models of vocal tract articulation during both speech and singing. This contrasts to the function of the right posterior lateral inferior cerebellum (Lobule VII) that was associated with word retrieval (Word retrieval from semantics. Time era 2002‐2006 and Word retrieval from semantics. Time era: 2007‐2011 sections above); and the very ventral and medial parts of lobule VIIIA that are activated when sensorimotor feedback is disrupted (see Auditory‐motor feedback during speech production. Time era 2007‐2011 section below).

Finally, the left putamen and thalamus were incorporated into a motor loop that passes activity from the SMA via the putamen to the thalamus and into the motor cortex (Bohland et al., 2010). This is consistent with the basal ganglia being involved in the innervations of vocal tract muscles (Brendel et al., 2010). A somewhat different view is that the basal ganglia (putamen and caudate) are involved in the timing, predictive coding and sequencing of events and this can be compensated for by a cerebellar-thalamic-pre-SMA pathway (Kotz et al., 2009; Kotz and Schwartze, 2010). There are also claims that the insula (rather than the SMA) activates the basal ganglia and cerebellum prior to motor output (Eickhoff et al., 2009). These connectivity studies showing the interactions between different regions are intriguing but further investigation is required to tie all sources of evidence together.

#### Overt articulation: 20 year summary

Producing the sounds of speech involves more than sensorimotor activity in the pre- and post-central regions (PrC and PoC in Table 2 and Fig. 3) that control the orofacial muscles. It also involves activation related to laryngeal activity, phonation and the voluntary control of breathing. A distinction has also been made between areas involved in motor execution (e.g. ACC in Table 1) and the cerebellum (CB in Table 2 and Fig. 3) and subcortical areas (PUT in Table 2 and Fig. 3) involved in the timing and control of motor activity.

### Auditory‐motor feedback during speech production

Articulation of speech produces sound for the listener that will also be heard by the speaker. During language acquisition, auditory processing of self-produced speech is used to tune motor production so that the produced auditory output matches the intended auditory output. In this sense, auditory feedback is useful for monitoring and correcting speech errors. Once speech is mastered, auditory feedback is less useful and we do not actively attend to the sound of our own voice. We may even inhibit auditory processing of the spoken response. Nevertheless, to anyone who has struggled to speak normally on a telephone line that delays the auditory feedback, it is clear that auditory feedback during speech production is not completely inhibited.

#### Auditory-motor feedback during speech production. Time era: 1992–1996

##### Novel findings

Auditory processing of self generated speech was inferred from observations that bilateral superior temporal gyri were activated during speaking aloud relative to making the articulatory movements of the same words without generating any sound (Price et al., 1996b). There were two qualifications to this observation: (a) superior temporal activation during self-vocalisation was less than that expected when perceiving another's voice (Hirano et al., 1996) and (b) left posterior temporal activation (in the planum temporale and perisylvian cortex) was observed during unvoiced syllable production when auditory processing was masked by low-intensity white noise (Paus et al., 1996b). To explain the activation in auditory processing areas during silent speech production, Paus et al. (1996b) emphasized that when we engage in motor activity, a discharge corollary to the motor command is sent from motor to sensory structures. Support for this hypothesis came from observations that the left posterior superior temporal cortex, extending into the left planum temporale, was activated when subjects imagined hearing another person's voice in the absence of any auditory input (McGuire et al., 1996a). Together these results suggested that auditory imagery during articulation resulted in left lateralized posterior temporal activation whereas auditory processing of the heard response after articulation resulted in bilateral superior temporal activation. This implies that left posterior temporal activation occurs prior to bilateral superior temporal activation but, to my knowledge, the differential timing of these responses has still not been tested.

#### Auditory-motor feedback during speech production. Time era: 1997–2001

##### Extending previous findings

Processing of self-produced vocalisations in bilateral auditory cortices was shown to be less than that of another's speech unless the speech fed back to the auditory system was altered to make it different from the articulated voice (Hirano et al., 1997). This suggests that, although auditory processing is normally less during articulation, it increases when the heard sounds are not expected. The response in the left planum temporale was again consistent with auditory imagery because it was observed when silently imagining speech (Shergill et al., 2001) or for recalling (imagining) the auditory relative to visual associations of a picture of a scene (Wheeler et al., 2000).

#### Auditory-motor feedback during speech production. Time era: 2002–2006

##### Extending previous findings

As shown previously, bilateral superior temporal activation was found to increase when there was a mismatch between the expected and actual auditory feedback (Hashimoto and Sakai, 2003; Fu et al., 2006). In the left planum temporale (previously associated with auditory imagery), activation was observed during subvocal articulation or the presentation of visual stimuli that had previously been experienced with auditory activity. For example, activation in the left planum temporale increased during the silence that followed familiar music even when there was no instruction to remember the music (Kraemer et al., 2005), when passively viewing finger tapping on the piano following keyboard training (Hasegawa et al., 2004), when producing rhythmic finger sequences that had been learnt with an auditory cue (Bengtsson et al., 2005) and when imagining heard speech, music or environmental sounds in the absence of sound (Aleman et al., 2005; Bunzeck et al., 2005; Zatorre and Halpern, 2005). These studies are consistent with the prior hypothesis that the left planum temporale is involved in auditory imagery and would explain why activation in the left planum temporale increased with the rate of covert (silent) speech production (Shergill et al., 2002), if we assume that auditory imagery (or inner speech) occurs automatically during covert speech production.

##### Novel findings

Auditory imagery during speech production might play an essential role in predicting the intended speech production, or even providing an internal model to which the auditory feedback should be matched (Heinks-Maldonado et al., 2005). There then needs to be a process by which the anticipated auditory response is integrated with the actual auditory response. This was addressed by Guenther et al. (2006) who proposed that there were “error cells” in the posterior superior temporal gyrus and planum temporale that respond when there is a mismatch between the intended/expected speech and the sound of the speech. The error signal is then fed back to the primary motor cortex to adjust the speech output so that it can be closer to that which was intended. Likewise, Guenther et al. (2006) proposed that there were “error cells” in the parietal (somatosensory) cortex that monitor the tactile and proprioceptive sensations.

#### Auditory-motor feedback during speech production. Time era: 2007–2011

##### Extending previous findings

In the bilateral superior temporal gyri associated with auditory processing, further studies showed that activation related to processing the sound of the speaker's own voice was less during the process of producing the speech than when hearing a recording of the spoken response (Ventura et al., 2009; Christoffels et al., 2011). This suppression of auditory processing was proportional to the quality of the feedback; consequently, superior temporal activation increased when speech was distorted (Christoffels et al., 2007; Tourville et al., 2008; Christofells et al., 2011; Zheng et al., 2010) or when auditory feedback was delayed (Takaso et al., 2010).

##### Novel findings

Six new findings emerged. First, in the left posterior planum temporale/temporoparietal area that previous studies had associated with the silent imagination of heard speech, activation was found to increase when speech production was more error prone due to interference or speaking in a second language (Hocking et al., 2009; Abel et al., 2009; Simmonds et al., 2011; Parker Jones et al., 2012). This is consistent with the mental imagery of the intended speech playing a role in monitoring speech production when it is error prone. Second, the left pars opercularis and left posterior superior temporal sulcus were reported to be more activated for making silent articulatory speech movements relative to silent nonverbal mouth movements, but the left posterior planum temporale was equally activated by verbal and nonverbal mouth movements (Price et al., 2011). This was interpreted in terms of the higher-order language areas that predict the auditory consequences of articulation. It also distinguishes the functional response in the left posterior planum temporale from that in the left pars opercularis and posterior superior temporal sulcus but does not elucidate the distinct contribution of each of these areas. Third, bilateral superior temporal activation was reported to be negatively correlated to that in the SMA (Van de Ven et al., 2009) which suggests that the role of the SMA in suppressing auditory feedback should be investigated. Fourth, activation in the right prefrontal cortex and rolandic cortical activity increased with bilateral superior temporal activation during distorted feedback (Tourville et al., 2008) which suggested a role for these areas in modulating subsequent speech output, or in resolving interference. Fifth, the posterior medial dorsal surface of the superior temporal gyri, including the planum temporale, were found to be activated during repetitive (silent) movements of the jaw and tongue as well as during auditory feedback (Dhanjal et al., 2008) and nonspeech vocal tract movements (Loucks et al., 2007). This highlighted a role for the posteromedial supratemporal plane in polysensory integration. Sixth, bilateral postcentral gyri were associated with somato-sensory feedback (Peschke et al., 2009; Zheng et al., 2010) and the consequences of this on compensatory speech motor commands were considered by Golfinopoulos et al. (2010) who found that jaw perturbations during speech increased activation in the left and right ventral motor cortex, inferior frontal cortex and inferior posterior cerebellum (lobule VIII).

#### Auditory-motor feedback during speech production: 20 year summary

Extrapolating from the findings so far, my speculation is that auditory monitoring of the spoken voice starts with an internal model of the intended speech which is generated in the core language areas (pOp and pSTS in Table 2 and Fig. 3). This results in auditory imagery (in STG and PT in Table 2 and Fig. 3). As the predictions become more precise, activity in the auditory cortices (L&R STG) decreases (with more activation when predictions are less precise).

### Visual word processing

Written words access the language system via the visual system. The sensory processing is therefore different from that required for the comprehension and production of auditory speech. The mapping of visual stimuli to articulation is also different from that involved in object naming. For example, words written in alphabetic script are composed of a limited number of visual features (letters) that provide clues to the pronunciation of the whole word. Phonology can therefore be retrieved from novel letter combinations that do not have learnt semantic associations (e.g. THACY). This means that there are infinitely more meaningful words that can be read than objects that can be named. Words can also be combined into sentences and narratives. The review of visual word processing below focuses only on the results of studies that aimed to find brain areas that are more activated by reading than either auditory word processing or visual object naming. The first section (Early visual word form processing. Time era: 1992–1996) focuses on brain areas activated by written words more than other types of stimuli. The second section (Dissociating neural pathways for mapping orthography to phonology) considers brain activation that might differ according to whether orthography is mapped to phonology at the lexical, sublexical or semantic level.

#### Early visual word form processing. Time era: 1992–1996

##### Novel findings

The early neuroimaging studies of reading suggested a special role for the left extrastriate visual cortex in visual word processing (Petersen et al., 1988, 1990, Petersen et al., 1989). Although the extrastriate cortex is clearly involved in orthographic (letter) processing (Pugh et al., 1996), subsequent studies emphasized the importance of three different regions in visual word form processing. The first was the left posterior middle/superior temporal gyrus which was more activated for reading aloud than viewing ‘false fonts’ (non-existent letter-like forms that controlled for visual input) and saying a single word (e.g. “crime” or “range”) to control for speech production (Howard et al., 1992; Small et al., 1996). The second was the left angular gyrus that was more activated for viewing words than pictures (Menard et al., 1996), and also the site of the “visual word form area” in the classical neurological model of reading (Dejerine, 1891; Geschwind, 1965). The third was the left ventral occipitotemporal cortex that was more activated by reading the Japanese script Kanji than Kana (Kiyosawa et al., 1995); and more activated when younger relative to older adults read English words (Madden et al., 1996).

Explanations for the inconsistent localization of visual word form processing focused on the experimental design and emphasized that activation changed with the task (Sergent et al., 1992; Price et al., 1994), the exposure duration of the stimuli (Price et al., 1994), their rate of presentation (Price et al., 1996d) and difficulties selecting a suitable baseline task because word-like stimuli automatically access the language system irrespective of the task (Sergent et al., 1992; Price et al., 1996c). In brief, subtle variations in experimental design influenced brain activity during reading tasks and it was therefore premature to associate specific processing functions with individual anatomical areas.

#### Early visual word form processing. Time era: 1997–2001

##### Extending prior findings

The involvement of the left occipitotemporal cortex in visual word form processing was not disputed (Fiez and Petersen, 1998; Fujimaki et al., 1999; Cohen et al., 2000; Hart et al., 2000; Dehaene et al., 2001; Leff et al., 2001). Meanwhile, reading-related activation in the left extrastriate cortex was attributed to early visual processing (Indefrey et al., 1997) and that in the posterior middle temporal and angular gyri was associated with semantic processing (Vandenberghe et al., 1996).

The strongest and most influential claim was that the left occipitotemporal cortex housed abstract representations of visual words (Cohen et al., 2000; Dehaene et al., 2001). This led to the left occipitotemporal cortex being labelled the ‘visual word form area’ (VWFA). Although damage to the left occipitotemporal cortex is known to impair reading (Leff et al., 2001), confusion and controversy emerged at the level of functional specialisation and anatomy. At the functional level, the abstract visual word processing claim was challenged by observations that activation for written words (that have abstract visual word form representations) was less than that for stimuli that don't have abstract word representations such as (a) unfamiliar pseudowords (Brunswick et al., 1999; Fujimaki et al., 1999; Tagamets et al., 2000; Xu et al., 2001) or (b) pictures of objects (Vandenberghe et al., 1996; Chee et al., 2000; Moore and Price, 1999). At the anatomical level, the left occipitotemporal activation associated with reading was located on the medial surface of the inferior temporal gyrus, at the boundary with the fusiform gyrus and at the junction between the occipital and temporal lobes. Hence it was referred to with multiple names: posterior inferior temporal, fusiform, occipitotemporal and the “VWFA”. Different sub-divisions of the left occipitotemporal reading area were also dissociated with different functional attributes (Moore and Price, 1999) leading to a situation where the same activation could be given different anatomical and functional labels.

#### Early visual word form processing. Time era: 2002–2006

##### Extending prior findings

Reports of activation during visual word form processing continued to focus solely on the role of the left occipitotemporal cortex which was also referred to as the left mid-fusiform gyrus and visual word form area (VWFA). All studies agreed that this area was consistently activated by visual word processing across languages and orthographies (e.g. Cohen et al., 2002; Turkeltaub et al., 2002; Fu et al., 2002; Price and Devlin, 2003; Reinholz and Pollmann, 2005; Vigneau et al., 2005). Activation was also reported to be higher for written words than spoken words (Booth et al., 2002a, 2002b; Cohen et al., 2002), written words than chequerboards or consonants (Cohen et al., 2002); and to be invariant to the spatial location of the stimuli (Cohen et al., 2002) or the case and font of the letters (Dehaene et al., 2002). The anatomical location of the visual word processing activation was also distinguished from other surrounding areas involved in single letter processing (Flowers et al., 2004) and amodal semantic processing (Nakamura et al., 2005; Cohen et al., 2004; Price and Mechelli, 2005).

Observations that left occipitotemporal activation was observed for pseudowords with increased activation as letter strings became more word-like (Binder et al., 2006) led to suggestions that learning to read tuned the receptive properties of the underlying neurons to combinations of letters (such as bigrams and trigrams) that are found within familiar words (Cohen and Dehaene, 2004; Binder et al., 2006). However, this perspective did not explain why left occipitotemporal activation was less for (a) words with high relative to low lexical frequency (Kuo et al., 2003; Kronbichler et al., 2004) or (b) familiar words than pseudowords (Mechelli et al., 2003; Kronbichler et al., 2004). To explain these “lexical familiarity effects”, Kronbichler et al. (2004) proposed that the left occipitotemporal cortex was specialised for extracting and storing abstract whole word patterns. According to this account, the amplitude of the activation increases with the difficulty encountered when matching a visual word form to its lexical representation (i.e. low > high frequency words; pseudowords > words). This lexical account can explain why left occipitotemporal activation is reduced by the repetition of a word (“sold–sold”) but not to a repetition to a pseudoword (“solst–solst”) but cannot explain why left ventral occipitotemporal activation was sensitive to sublexical similarities between words (e.g. “corner–corn”) that had different lexical representations (Devlin et al., 2006).

A third perspective was that the same left occipitotemporal neurons were activated by object recognition and colour naming tasks and therefore the function of this region was not specific to either letter combinations or whole word forms (Price and Devlin, 2003; Joseph et al., 2003, 2006). Instead, the function appeared to be one that integrated visual information with higher-level processing (Price and Devlin, 2003; Price and Friston, 2005; Vigneau et al., 2005; Devlin et al., 2006). This would explain why activation in this region was sensitive to lexicality effects (Kronbichler et al., 2004) and prior experience (Dehaene et al., 2001). It also explains why the left fusiform responses to letters relative to unfamiliar shapes were task dependent (Pernet et al., 2005) and why left occipitotemporal responses to novel orthographic stimuli changed with the type of training experienced (Sandak et al., 2004; Xue et al., 2006). For example, Xue et al. (2006) found that activation increased after phonological and semantic training but decreased after visual form training. These findings highlighted the influence of higher-level phonological and semantic associations on left occipitotemporal activation.

#### Early visual word form processing. Time era: 2007–2011

##### Extending prior findings

There was a continued focus on the role of the left ventral occipitotemporal cortex in visual word form recognition (Wandell, 2011). As previously documented, activation for processing word and word-like stimuli that have access to learnt abstract visual form representations was observed relative to unfamiliar nonword stimuli matched for visual complexity (Liu et al., 2008), irrespective of the hemifield of presentation (Woodhead et al., 2011a) and the physical form that the words were presented in Qiao et al. (2010) and Kronbichler et al. (2009). More details of the perceptual feature-to-whole word gradient along the posterior–anterior axis of the left occipitotemporal cortex were described for both alphabetic texts (Vinckier et al., 2007; Brem et al., 2010; Nosarti et al., 2010; Seghier and Price, 2011; Woollams et al., 2011) and Chinese/Korean texts (Chan et al., 2009).

Several studies also replicated prior observations that there was remarkable similarity in the response to visual form processing of letters, words and objects (Eddy et al., 2007; Wright et al., 2008; Turkeltaub et al., 2008; Burgund et al., 2009; Kherif et al., 2011; Shinkareva et al., 2011). The only studies that claimed to have found greater activation for words than pictures in the left ventral occipitotemporal cortex did not control for semantic and phonological attributes of the stimuli (Baker et al., 2007; Szwed et al., 2011) and used low level perceptual tasks such as the one back task (is the stimulus the same as the previous stimulus) that permit stimulus specific strategies. For example, greater activation for words, particularly in the anterior fusiform part of the ventral occipitotemporal cortex (Szwed et al., 2011) that has previously been associated with semantic processing (see Speech comprehension section above), may reflect the use of a semantic strategy for words that were not used for pictures.

The effect of learning/experience on left ventral occipitotemporal activation was reported in two contrasting ways. In the early stages of children or adults learning to read, left ventral occipitotemporal activation increased with learning (Brem et al., 2010; Dehaene et al., 2010b) and this correlated with the rate of improvement in word recognition (Ben-Shachar et al., 2011). However, in skilled readers, activation decreased with experience/exposure to the same stimuli (Wong et al., 2009; Xue et al., 2010; Song et al., 2010a, 2010b; Xue and Poldrack, 2007) and for stimuli with high relative to low orthographic familiarity (Kronbichler et al., 2007; Bruno et al., 2008) and lexical frequency (Kronbichler et al., 2007). These experience-dependent effects illustrate that the response in the left ventral occipitotemporal cortex changes with learning but the interpretation of the learning effect was debated.

One interpretation is that, during the course of learning to read, the response properties of the left ventral occipitotemporal cortex (or left mid-fusiform) become selective to learnt orthographic representations (Dehaene and Cohen, 2007, 2011) with orthographic familiarity effects observed independent of phonological or semantic familiarity (Kronbichler et al., 2007). Some authors further argued that specialisation for orthographic processing is at the whole word (lexical) level (Kronbichler et al., 2007, 2009; Glezer et al., 2009; Schurz et al., 2010) but agreed that orthographic processing in the left ventral occipitotemporal cortex is a precursor for mapping visual forms onto meaning and articulatory representations (Yarkoni et al., 2008).

A second perspective is that the left occipitotemporal cortex is involved in the perceptual processing of generic visual features that are present to varying degrees in all visual stimuli including words, objects, letters and faces (Barton et al., 2010; Mei et al., 2010; Braet et al., 2011; Reinke et al., 2008). Within this framework, evidence was presented to support a role for the left ventral occipitotemporal cortex in (a) generic visual memory (Mei et al., 2010); (b) convergence of features (Rauschecker et al., 2011); (c) high spatial frequencies that may bias the lateralization of processing irrespective of its higher-order properties (Woodhead et al., 2011b); and (d) attention to spatial and feature processing that is related to activity in dorsal parietal regions (Vogel et al., 2011). Although left occipitotemporal activation is not specific to written words in these accounts, specialisation for words arises in the unique network of brain regions that are activated during the word condition (Reinke et al., 2008). In other words, the process of learning to read integrates generic visual processing with higher-order language areas and there is no need for brain areas that are specialised for orthographic processing.

A complementary perspective is that the left ventral occipitotemporal cortex contributes to written word recognition by integrating bottom up (feed forward) generic visual processing with top-down influences from phonological, and semantic areas (Cai et al., 2010; Hellyer et al., 2011; Price and Devlin, 2011; Wang et al., 2011; Woodhead et al., 2011a). After learning to read, these top-down influences are generated automatically (irrespective of task) in response to written words, but their strength can also be modulated by task and attention (Guo and Burgund, 2010; Borowsky et al., 2007; Hellyer et al., 2011; Twomey et al., 2011; Yoncheva et al., 2010; Woodhead et al., 2011a). This integration of visual, semantic and phonological information is not unique to written words but is required by other tasks, particularly object naming. The same left ventral occipitotemporal site also appears to function as a multi-modal integration area in the absence of visual inputs as indicated by its response during non-visual braille reading in congenitally blind participants (Büchel et al., 1998; Reich et al., 2011).

This interactive account of left ventral occipitotemporal cortical responses can explain a wide range of observations including increased activation to orthographic forms when learning to read and decreased activation as reading becomes easier (Price and Devlin, 2011). It also explains why left occipitotemporal activation is sensitive to the left-right orientation of single letters and words but not to pictures (Dehaene et al., 2010a; Pegado et al., 2011) in terms of the learnt relationship between the visual form and higher-level language associations (which are orientation-specific for letters/words but not for objects). The early influence of language on visual word processing in the left ventral occipitotemporal cortex is consistent with observations that (a) the response to written words in a left-lateralized inferior frontal region (pars opercularis) peaks at the same time as that in the left ventral occipitotemporal cortex (Cornelissen et al., 2009); and (b) activation during picture naming or reading aloud is reduced when the target stimulus to be named is preceded by an unconscious masked prime that has the same name as the target but a different physical form (Eddy et al., 2007) as when a word is primed by a picture or a picture is primed by a word (Kherif et al., 2011).

Finally, the degree to which the response in the ventral occipitotemporal cortex was left lateralized for words was found to correlate with the degree to which inferior frontal activation was left lateralized during word generation (Cai et al., 2010). The determinants of lateralization also varied with the subregion of occipitotemporal cortex tested (Seghier and Price, 2011). In the posterior subregion, lateralization depended on the spatial frequency of the visual inputs. In the anterior subregion, lateralization depended on the semantic demands of the task. In the middle part that has been the focus of the discussion above, lateralization was explained by decreased activation in right occipitotemporal cortex as visual expertise increased. Therefore, left lateralized activation in the ventral occipitotemporal cortex depends on the subregion tested and does not necessarily indicate a specialisation for orthography in left ventral occipitotemporal cortex.

#### Early visual word form processing: 20 year summary

There is no doubt that an extensive region of the ventral occipitotemporal cortex is involved in skilled reading. Within this region, posterior areas are involved in visual feature extraction and more anterior areas are involved in lexico-semantic processing of the whole word. How the response properties in this system differ for written words and other stimuli is still a matter of debate.

### Dissociating neural pathways for mapping orthography to phonology

This section considers studies that have attempted to dissociate neural pathways for converting spelling (orthography) to sound (phonology) via sublexical, lexical and semantic routes. The sublexical route involves assembling the phonology associated with the whole word from its sublexical parts (sublexical orthographic to phonological conversion). The lexical route involves retrieving phonology directly from the orthography of the whole word. The semantic route involves retrieving phonology from the semantic properties of the word (similar to picture naming).

The sublexical route is particularly important when reading new words (e.g. for pseudowords like THACY). In contrast, the lexical or semantic routes are particularly important when the sublexical spelling to sound relationships are “inconsistent” with the whole word representation (e.g. for reading irregularly spelled words like YACHT). One approach for segregating sublexical and lexical reading routes has therefore been to contrast activation for reading pseudowords with activation for reading words with irregular spellings. Another approach has been to compare reading of different alphabetic or nonalphabetic scripts that differ in the depth and consistency of their phonological clues. For example, the relationship between orthography and phonology is most consistent in Italian and least consistent in Chinese. Conversely, Chinese relies more heavily on lexical knowledge than English because Chinese is a logographic orthography that has weak phonological clues. Japanese is particularly interesting because the same words can be written in different scripts with different properties. For example, the Japanese script Kana can be read on the basis of sublexical phonological clues whereas the Japanese script Kanji must be processed at the level of morphemes (the smallest unit of meaning). Comparison of activation for different scripts (Italian versus English; English versus Chinese; Kanji versus Kana) can therefore provide clues to the neural basis of different reading pathways.

#### Dissociating neural pathways for mapping orthography to phonology. Time era: 1992–1996

##### Novel findings

A comparison of activation for reading the Japanese scripts Kanji and Kana found greater activation for Kanji in the posterior part of the primary visual cortex but did not find any areas that were significantly more activated for Kana (Kiyosawa et al., 1995). A dissociation between the scripts was, nevertheless, observed at the level of functional connectivity because, within the common set of areas that were activated for Kanji and Kana, functional connectivity was stronger in ventral reading areas for Kanji reading, and in dorsal reading areas for Kana reading (Kiyosawa et al., 1995). This study therefore provided evidence that morphemic reading could be differentiated from sequential (sublexical) reading. A less optimistic start was reported for the comparison of word and pseudoword reading. A double dissociation in brain activation proved to be elusive because, throughout the reading system, activation for pseudowords was greater than that for words (Price et al., 1996a). The interpretation for this was that activation was higher when the links between orthography and phonology were unfamiliar or unsuccessful (pseudoword reading) compared to when they were familiar and successful(real words). Critically, however, there was no report of any study that directly compared activation for reading pseudowords with activation for reading irregularly spelled words.

#### Dissociating neural pathways for mapping orthography to phonology. Time era: 1997–2001

##### Extending prior findings

Several studies compared activation for familiar words and unfamiliar pseudowords (Herbster et al., 1997; Rumsey et al., 1997; Fiez and Petersen, 1998; Hagoort et al., 1999; Mechelli et al., 2000; Pugh et al., 2000, 2001; Tagamets et al., 2000; Xu et al., 2001; Bokde et al., 2001). Overall, there was a general agreement that a common neural network was activated by words and pseudowords with the most consistent difference between word types being greater activation for pseudowords, particularly in the left posterior inferior frontal cortex (Herbster et al., 1997; Hagoort et al., 1999; Fiez and Petersen, 1998; Haist et al., 2001; Xu et al., 2001). One interpretation of this effect was that pseudowords increase the demands on sublexical conversion of orthography to phonology (Hagoort et al., 1999; Pugh et al., 1996). The alternative interpretation was that pseudoword reading was more difficult. Fiez and Petersen (1998) and Fiez et al. (1999) demonstrated this by showing that left posterior inferior frontal activation was also higher for reading words with irregular spellings (e.g. KNIFE) than regular spellings (e.g. BROOM) and was proportional to response times.

##### Novel findings

One study found that reading aloud words with irregular spellings increased activation in the left anterior ventral occipito-temporal cortex relative to reading aloud pseudowords (Herbster et al., 1997). As irregular word reading is reliant on lexico-semantic processing, the result is consistent with prior and new claims (Kiyosawa et al., 1995; Tokunaga et al., 1999) that ventral parts of the reading system were more activated for semantic reading (Kanji) than sublexical reading (Kana).

Plausibly, other studies of word and pseudoword reading in alphabetic scripts did not identify increased activation in the left anterior occipito-temporal cortex because they didn't specifically assess activation for words with irregular spellings or didn't scan the anterior parts of the left occipitotemporal cortex/fusiform which lie on the ventral surface of the brain and are therefore often excluded from the field of view. Evidence that a more posterior left ventral occipitotemporal area was involved in lexical compared to sublexical reading came from the observation that activation in this area was stronger in English than Italian readers, (Paulesu et al., 2000), particularly when the stimuli were pseudowords. This was interpreted in terms of reading strategy differences because Italian is a regularly spelled language and therefore sublexical links between orthography and phonology are reliable. In contrast, English is an irregularly spelled language and therefore lexical influences are always in place, even during pseudoword reading.

Lexical and sublexical orthographic processing were also dissociated at the level of the functional interactions between shared processing areas. Specifically, Bokde et al. (2001) demonstrated that, relative to pseudowords, words increased the functional coupling between left occipitotemporal cortex and the left ventral inferior frontal areas associated with semantic processing while decreasing the coupling between the left occipitotemporal cortex and inferior frontal regions associated with phonological processing. Pugh et al. (2000, 2001) also distinguished different reading pathways from the occipitotemporal cortex with: a ventral pathway sustaining fast, fluent word recognition, and a dorsal pathway via the temporoparietal cortex supporting the analytic processing required for learning to integrate orthographic with phonological and semantic features of printed words. Overall, these findings were consistent with cognitive models of reading, where multiple pathways are activated by word-like stimuli with the level of activation in each pathway depending on the familiarity of the stimulus and the consistency between the orthography (letters) and phonology (sounds). There was also evidence that there might be other reading pathways in the right hemisphere (Pugh et al., 1997; Hart et al., 2000; Mayall et al., 2001) particularly for reading in Chinese (Tan et al., 2001a, 2001b).

#### Dissociating neural pathways for mapping orthography to phonology. Time era: 2002–2006

##### Extending prior findings

In a meta-analysis of 35 previous neuroimaging studies of reading, Jobard et al. (2003) dissociated two routes for reading: (a) a lexicosemantic route involving the left anterior ventral occipitotemporal cortex (basal temporal language area), the posterior part of the middle temporal gyrus, and the triangular part of inferior frontal gyrus; and (b) direct links between orthography and phonology involving left lateralized superior temporal areas, supramarginal gyrus, and the opercular part of the inferior frontal gyrus; all regions that are also involved in the articulatory loop component of short term memory which is required for phonological decisions on pseudowords.

The involvement of an anterior fusiform/ventral occipitotemporal area in the semantic route was confirmed in a study showing that regional activation in the anterior ventral occipitotemporal cortex and the left ventral inferior frontal cortex was stronger for irregularly spelt words than pseudowords or words with regular spellings (Mechelli et al., 2005). Moreover, the functional connectivity between these two areas was also stronger for irregular words than pseudowords (Mechelli et al., 2005). Other regions associated with the semantic reading route were the left posterior temporal and parietal cortices, where activation was higher for familiar words than pseudowords (Fiebach et al., 2002; Binder et al., 2003, 2005; Jobard et al., 2003; Ischebeck et al., 2004; Vigneau et al., 2005; Borowsky et al., 2006) and for words with irregular compared to regular spellings (Senaha et al., 2005; Lee et al., 2004; Frost et al., 2005). Because these areas were also more activated by semantic than phonological decisions, their role in irregular word reading was again attributed to increased demands on semantic processing when sublexical access to phonology was not possible (McDermott et al., 2003; Price and Mechelli, 2005; Booth et al., 2006).

While irregular word reading was associated with semantic activation, it also became evident that there was a correspondence between the areas that are more activated for reading pseudowords than real words and the areas activated by phonological relative to semantic decisions. Specifically, the left opercular part of the inferior frontal gyrus, left precentral gyrus, insular cortex, supramarginal gyrus and superior temporal areas that were more activated for phonological than semantic decisions on written words (McDermott et al., 2003; Booth et al., 2006; Price and Mechelli, 2005) corresponded to activations that were stronger for reading pseudowords than words (Fiebach et al., 2002; Binder et al., 2003, 2005; Mechelli et al., 2003, 2005; Jobard et al., 2003; Owen et al., 2004; Xiao et al., 2005; Dietz et al., 2005; Borowsky et al., 2006), for Japanese words presented in Kana relative to Kanji (Thuy et al., 2004); for reading Spanish than English (Meschyan and Hernandez, 2006), for reading words than naming pictures (Price et al., 2006) and for unfamiliar than familiar words (Fiebach et al., 2002; Ischebeck et al., 2004).

An appealing interpretation of increased phonological activation for pseudowords compared to words was that it reflected the demands on accessing phonology from sublexical orthographic codes. Indeed, Bitan et al. (2005) noted that left posterior inferior frontal activation was greater for novel words in an artificial script after new letter decoding instructions had been learnt. However, as pointed out previously by Fiez et al. (1999), Binder et al. (2005) noted that there were no areas where activation corresponded to increasing demands on phonological decoding (i.e. irregularly spelled words < regularly spelled words < pseudowords) but instead activation depended on overall response times (regularly spelled words < irregularly spelled words < pseudowords). Xiao et al. (2005) also pointed out that left inferior frontal activation for pseudowords was not necessarily reflective of grapheme-to-phoneme conversion because this area was more activated by auditory lexical decisions on pseudo Chinese words than real Chinese words, even though the auditory task did not involve grapheme-to-phoneme conversion. Likewise, the demands on sublexical phonological processing do not easily explain why activation in the left inferior frontal cortex was higher for pseudohomophones that sound like familiar words (e.g. BRANE) than pseudowords (e.g., BLINT) that don't sound like familiar words (Edwards et al., 2005).

Overall, definitive interpretations of word and pseudoword activation differences were difficult because words and pseudowords differ in more than one way (e.g. visual familiarity, access to semantics and phonological decoding) and because differences in activation are only relative (rather than absolute) within areas that are commonly activated by a range of stimuli and tasks (Jobard et al., 2003; Mechelli et al., 2003).

#### Dissociating neural pathways for mapping orthography to phonology. Time era: 2007–2011

##### Extending previous findings

Studies comparing activation for reading scripts with consistent (or transparent) and inconsistent (opaque) orthographies (Matsuo et al., 2010; Hu et al., 2010; Das et al., 2011) reported left inferior parietal or posterior superior temporal activation for more consistent orthographies (Italian and Hindi versus English; and English versus Chinese) and left middle frontal activation when phonological information was minimal or conflicting (Chinese versus English and Kanji versus Chinese). Nevertheless, predominantly common activation across all scripts and the task dependent nature of the script differences (Ino et al., 2009; Liu et al., 2009) make it difficult to dissociate the anatomical components of different reading pathways on the basis of script differences alone.

Studies comparing word and pseudoword reading within script provided further evidence that the left posterior occipitotemporal cortex was more activated by pseudowords than real words (Levy et al., 2009; Nosarti et al., 2010; Woollams et al., 2011) while the left anterior occipitotemporal cortex was more activated by irregular (inconsistently spelled) words than regular words (Nosarti et al., 2010; Graves et al., 2010a). With respect to other regions in these pathways, semantic reading in the anterior ventral occipitotemporal cortex was again associated with activation in the ventral inferior frontal cortex (Graves et al., 2010a), non-semantic serial decoding in the left supramarginal gyrus (Graves et al., 2010a) was associated with auditory short term memory and more dorsal parietal activation was associated with visual attention (Cohen et al., 2008).

##### Novel findings

A rather different dual route neural model of reading was proposed by Levy et al. (2009) who suggested that the left posterior occipitotemporal cortex was involved in sublexical processing and was only necessary for pseudoword reading. In contrast, familiar words could be read without left occipitotemporal activation by virtue of direct connectivity between occipital and parietal regions. Some support for the hypothesis that not all reading pathways involved the left ventral occipitotemporal cortex was later reported by Levy et al. (2008, 2009) and Richardson et al. (2011) who found evidence for links between inferior occipital and posterior superior temporal areas that were independent of activity in the left ventral occipitotemporal cortex. These studies therefore raise the interesting possibility that the left occipitotemporal cortex is not essential for accessing phonology from orthography. Future functional imaging studies are now required to test whether patients with left occipitotemporal damage who are able to read short familiar words activate the left occipital and parietal areas proposed by Levy et al. (2009) and/or the left occipital and superior temporal areas proposed by Richardson et al. (2011).

The availability of different reading routes, for the same word stimuli, offers the potential for inter-subject variability in which routes are most strongly activated. This has been demonstrated in several studies. For example, Seghier et al. (2008) found that, when reading a single set of familiar words, some skilled readers showed more activation in the anterior occipitotemporal–inferior frontal semantic pathway while other skilled readers showed more activation in a left posterior occipitotemporal–right inferior parietal non-semantic pathway. This and other studies (Bolger et al., 2008a, 2008b; Levy et al., 2009) have also shown that the effect of spelling-sound consistency on brain activation depends on reading skill. Another important result for disambiguating the function of different reading areas was the observation that producing the visual forms associated with articulated words (i.e. spelling to dictation) activates the left ventral occipito-temporal cortex and left pars opercularis that also sustain reading (Purcell et al., 2011; Rapp and Lipka, 2011, Rapp and Dufor, 2011). Thus these areas are activated by both the translation of visual forms to articulation as well as the translation of articulation to visual forms.

#### Dissociating neural pathways for mapping orthography to phonology: 20 year summary

The clearest dissociation so far is between a lexico-semantic reading route that integrates the left ventral occipitotemporal cortex (LvOT in Table 2 and Fig. 3) with the left ventral inferior frontal gyrus, and a non-semantic phonological decoding route that links the superior temporal and ventral inferior parietal cortices to the dorsal precentral gyrus. Preliminary evidence suggests that the point of initial divergence is prior to activation in the ventral occipitotemporal area that some refer to as the visual word form area. However, it remains unclear how these pathways overlap and dissociate in the rest of the neural system for reading. My prediction is that there are multiple brain regions and multiple interconnections that underlie the reading system and these provide many possible reading pathways that are not yet appreciated in cognitive models.

## Conclusions

In the words of Raichle (1996): “*Modern functional brain imaging with PET and fMRI provides a new perspective on the organization of language in the human brain; a better definition of the distributed nature of the brain circuits involved, an appreciation of the flexibility of these circuits in adapting to the different aspects of speech production, an identification of areas not previously associated with the cognitive aspects of language, and a new understanding of the implications of specific brain lesions.*”

Indeed, our understanding of the functional anatomy of language has come a long way since the neurological model of Broca's and Wernicke's areas that dominated the field 20 years ago. For example, we now appreciate the importance of the cerebellum for word generation (Ackermann et al., 1997) and the involvement of the basal temporal language area, anterior cingulate and left inferior prefrontal cortex in a range of different language tasks (Chertkow and Murtha, 1997). In contrast there are other areas where activation was predicted by lesion studies but not observed during functional imaging studies, such as the absence of activation in the left angular gyrus during reading aloud (Ackermann et al., 1997; Price, 2000).

A striking feature is that the same conclusions have been produced over and over again. Although this results in repetitive reading, it is important for validating the findings and demonstrating the remarkable consistency of the functional anatomy across individuals and studies. Yes, there are interesting and relevant sources of inter-subject variability but these are small relative to the consistent effects. The next 20 years will need to focus on understanding how different regions interact with one another and how specialisation for language arises at the level of distinct patterns of activation in areas that participate in many different functions.

## Figures and Tables

**Table 1:** Definitions of the terminology used in Table 2.

Auditory processing of speech and nonspeech sounds	
Acoustic processing	Response to hearing all types of auditory stimuli.
Rapid transition	When auditory stimuli are changing rapidly.
Acoustic complexity	Response that increase as the auditory input becomes more complex.
Familiar sounds	Response that increase when sounds are familiar (like learnt speech).
Auditory imagery	Hearing familiar sounds in the head, in the absence of auditory inputs.
Short term memory	Maintaining auditory imagery in the absence of auditory inputs.
Speech selective auditory processing (= phonological processing)	
Speech sounds	Responses that are greater for speech sounds than other types of sound.
Articulatory recoding	Linking speech sounds to their articulatory associations.
Speech comprehension (semantic and syntactic processing)	
Accessing semantics	Accessing the meaning or interpretation of a word or sentence.
Semantic associations	Meanings that are similar to one another or concepts that occur together.
Influence of context	When the meaning of a word depends on the meaning of other words.
Integrating/predicting	Guessing meaning on the basis of the general multimodal context.
Sentence meaning	Sentence meaning that is more than the sum of the component words.
Narratives	A set of sentences whose meanings integrate into on a coherent story.
Selection/retrieval	Finding a concept from many possibilities, using a particular criteria.
Word retrieval	
Word selection	Finding words from multiple competing possibilities for the same concept.
Word suppression	Suppressing the retrieval of unintended words.
Semantics to phonology	Linking semantic processing with articulatory planning.
With minimal semantics	Finding words when semantic content is limited (rather than competing).
Covert (silent) articulatory planning for the production of speech sounds (phonological output)	
General action selection	Selecting motor commands for what to do next, from alternative options.
Sequencing motor plans	Ordering the different components of complex motor commands.
Orofacial motor planning	Motor commands that specifically control mouth and face movements.
Auditory expectation	Internal representation of the sounds that articulations should produce.
Overt articulation (i.e. speaking aloud)	
Motor execution	Initiating and implementing the selected motor commands.
Orofacial motor activity	Motor activity that controls mouth and face movements.
Timing of motor output	Ensuring that the timing of motor execution occurs as planned.
Breathing control	Motor activity that co-ordinates breathing with orofacial movements.
Auditory and motor feedback during speech production	
Auditory processing	Auditory response to sounds produced by orofacial motor activity.
Auditory imagery	Auditory response that is anticipated from the motor activity.
Auditory expectation	Representations that the generate the prediction of auditory feedback.
Visual word form processing	
Visual word forms	Responses that are greater for written words than other visual forms.
Familiar visual forms	Visual forms that have semantic and articulatory associations.
Visual imagery	Imagining familiar visual forms, in the absence of visual inputs.
Visual expectation	Representation of orthography associated with articulation (e.g. for spelling).
Dissociating neural pathways for mapping orthography onto phonology	
Sublexical reading	Mapping sublexical spellings to sublexical sounds (e.g. for pseudowords)
Lexical reading	Mapping whole word spellings to whole word sounds (known words only)
Semantic reading	Mapping semantics to whole word sounds (e.g. for irregularly spelt words)

**Table 2:**
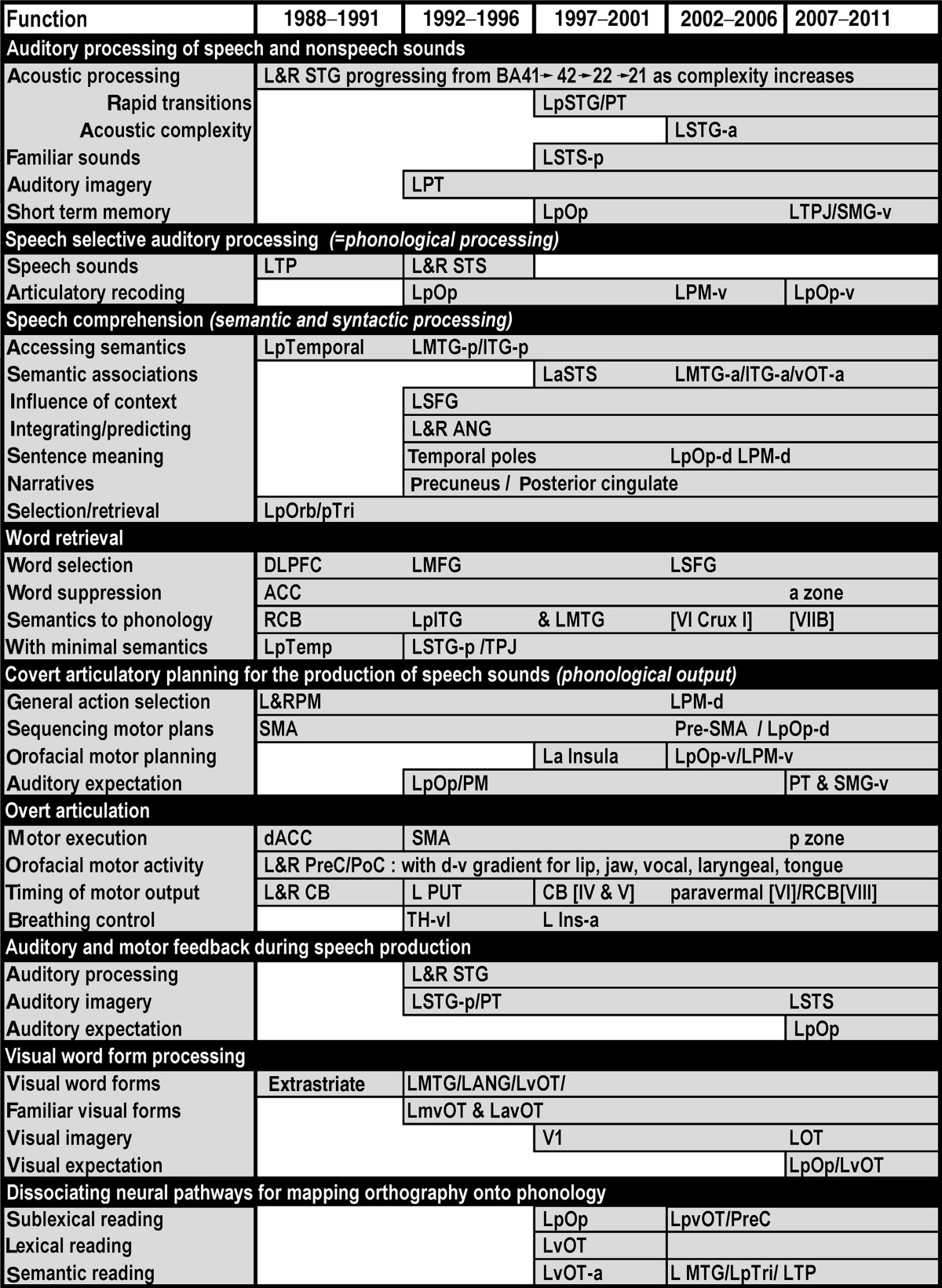
Historical perspective on the emergence of function-to-structure mappings (see Table 3 for key to abbreviations).

**Table 3:** Anatomical regions corresponding to the abbreviations in Table 2.

ACC-a zone	Anterior cingulate, anterior zone
ACC-p zone	Anterior cingulate, posterior zone
ANG	Angular gyrus
CB [IV and V]	Cerebellum: bilateral, medial, anterior (lobules IV and V)
CB [VI/VIIB]	Cerebellum: right lateral posterior (lobules VI and VIIB)
CB [VI] medial	Cerebellum: bilateral, medial, superior (paravermal lobule VI)
CB [VIII]	Cerebellum: right inferior posterior (lobule VIIIA)
Ins-a	Insula — anterior part at the junction with frontal operculum
ITG-p	Inferior temporal gyrus — posterior part
ITG-a	Inferior temporal gyrus — anterior part
MFG	Middle frontal gyrus at the junction with the inferior frontal sulcus
MTG-p	Middle temporal gyrus — posterior part
MTG-a	Middle temporal gyrus — anterior part
PM-d	Premotor cortex — dorsal
PM-v	Premotor cortex — ventral
pOP	Pars opercularis in the inferior frontal cortex (BA 44/Broca's area)
pOp-d	Pars opercularis — dorsal
pOp-v	Pars opercularis — ventral
pOrb	Pars orbitalis (ventral anterior inferior frontal cortex)
PreC/Poc	Precentral and postcentral (rolandic cortex)
Pre-SMA	Anterior to the supplementary motor cortex
PT	Planum temporale (on supratemporal plane/dorsal surface of pSTG)
pTri	Pars triangularis (BA 45, anterior to Broca's area)
PUT	Putamen
SFG	Superior frontal gyrus
SMA	Supplementary motor cortex
SMG-v	Supramarginal gyrus — ventral
STG-a	Superior temporal gyrus — anterior
STG-p	Superior temporal gyrus — posterior
STS-a	Superior temporal sulcus — anterior
STS-p	Superior temporal sulcus — posterior
TH – vl	Thalamus — ventral lateral
TPJ	Temporo-parietal junction
vOT	Ventral occipito-temporal cortex around the occipito-temporal sulcus
vOT-a	Ventral occipito-temporal-anterior
vOT-p	Ventral occipito-temporal‐posterior

**Table 4:** Consistent structure-to-function mappings in language studies.

ACC-a zone	Suppressing the production of unintended words
ACC-p zone	Motor execution (suppressing unintended motor activity)
ANG	Integrating/predicting semantics
CB [IV and V]	Silent articulatory planning
CB [VI/VIIB]	Retrieving words for speech production
CB [VI] medial	Timing of motor output
CB [VIII]	Sensitive to timing of auditory inputs and motor activity
Ins-a	Control of breathing during production of speech
ITG-p	Accessing semantics during word production tasks
ITG-a	Semantic associations
MFG	Retrieving words for speech production
MTG-p	Accessing semantics
MTG-a	Semantic associations
PM-d	General action selection (i.e. not specific to speech articulation)
PM-v	Orofacial motor planning (articulatory recoding)
pOp	Short term memory and integrating inputs, expectations, meaning
pOp-d	Sequencing subsequent motor activity
pOp-v	Articulatory recoding (orofacial motor planning)
pOrb	Selection/retrieval or semantic concepts/words
PreC/Poc	Orofacial motor activity (d-to-v: lips, jaw, laryngeal, tongue)
Pre-SMA	Sequencing motor plans (not specific to articulation)
PT	Acoustic processing/auditory imagery/auditory expectations
pTri	Semantic decisions/semantic reading
PUT	Timing of motor output
SFG	Semantic/word selection depending on semantic context
SMA	Sequencing execution of motor movements (speech and fingers)
SMG-v	Articulatory loop/auditory expectations
STG-a	Early auditory processing of complex sounds
STG-p	Auditory processing/word retrieval with minimal semantics
STS-a	Semantic associations
STS-p	Integrating familiar sounds, articulation and meaning
TH v-l	Control of breathing during speech production
TPJ	Auditory short term memory/word retrieval with minimal semantics
vOT	Linking visual forms to the semantic system
vOT-a	Accessing semantics from visual forms
vOT-p	Early visual processing of sublexical forms.
